# *ID3*-depleted human induced pluripotent stem cell–derived neural stem/progenitor cells promote neurorepair

**DOI:** 10.4103/NRR.NRR-D-24-01535

**Published:** 2025-09-03

**Authors:** Jia-Di Lin, Ruba Hammad, Prateek Kumar, Pedro Manzitti, Kexin Wu, Jamal Alzubi, Andreas Vlachos, Toni Cathomen, Armin Blesch, Yu-Hsuan Chu, Christian Schachtrup

**Affiliations:** 1 Institute of Anatomy and Cell Biology, Faculty of Medicine, University of Freiburg, Freiburg, Germany; 2 Faculty of Biology, University of Freiburg, Freiburg, Germany; 3 Freiburg iPS Core, Institute for Transfusion Medicine and Gene Therapy, Medical Center - University of Freiburg, Freiburg, Germany; 4 Department of Neurosciences, Center for Neural Repair, University of California, San Diego, La Jolla, CA, USA and VA San Diego Healthcare System, San Diego, CA, USA; 5 Center for Chronic Immunodeficiency, Faculty of Medicine, University of Freiburg, Freiburg, Germany; 6 Department of Haematooncology, Faculty of Medicine, University of Ostrava, Ostrava, Czech Republic; 7 Department of Neuroanatomy, Institute of Anatomy and Cell Biology, Faculty of Medicine, University of Freiburg, Freiburg, Germany; 8 BrainLinks–BrainTools Center, University of Freiburg, Freiburg, Germany; 9 Center for Basics in NeuroModulation, Faculty of Medicine, University of Freiburg, Freiburg, Germany

**Keywords:** astrocyte, CRISPR-Cas9, extracellular vesicles, fibrinogen, human iPSC-derived neural stem/progenitor cell, inhibitor of DNA binding 3 (ID3), microRNA, nerve regeneration, neurite outgrowth, spinal cord

## Abstract

Human induced pluripotent stem cell–derived neural stem/progenitor cells are used in cell-replacement and regenerative therapeutic strategies after traumatic central nervous system injury. Traumatic injury alters the host microenvironment, which in turn affects the functionality of transplanted human neural stem/progenitor cells and potentially limits their benefits for neurorepair. However, the underlying mechanisms through which the host environment alters the fate and functionality of transplanted human neural stem/progenitor cells remain poorly understood. Here, we showed that massive deposition of blood-derived fibrinogen in a mouse model of spinal cord injury contributed to an altered lesion environment. Fibrinogen promoted human neural stem/progenitor cell differentiation into reactive astrocytes by activating the BMP receptor signaling pathway and inducing of the transcriptional regulator inhibitor of DNA binding 3. *ID3*-depleted human neural stem/progenitor cells, generated by CRISPR/Cas9-mediated genome editing, reduced astrocyte formation in response to astrogenic stimuli. Instead, *ID3*-depleted human neural stem/progenitor cells had a bipolar, immature glial progenitor cell phenotype. These modified cells secreted extracellular vesicles with a distinct miRNA profile that enhanced neurite outgrowth. We conclude that targeting inhibitor of DNA binding 3 in human neural stem/progenitor cells can beneficially modulate their functionality and cell fate in the injured central nervous system toward glial progenitor cells, potentially enhancing their capacity to promote central nervous system repair.

## Graphical abstract

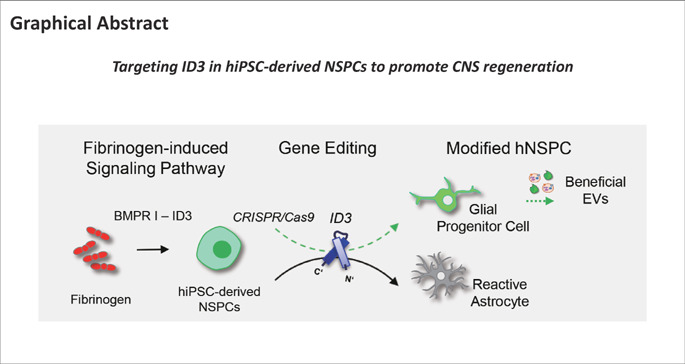

## Introduction

Traumatic central nervous system (CNS) injury results in the disturbance of neural circuitry through disruption of axonal pathways, neuronal damage or loss, and astrocyte scar formation (Bradbury and Burnside, 2019; Katoh et al., 2019; Zheng and Tuszynski, 2023). Endogenous or transplanted neural stem/progenitor cells (NSPCs) are a promising therapeutic approach to promoting repair after traumatic CNS injury (Zheng and Tuszynski, 2023; Hassan et al., 2024). Strategies to regulate the fate of NSPCs might be a key for CNS injury repair (Schachtrup, 2022; Zhang et al., 2022; Malik et al., 2023). Human induced pluripotent stem cells (hiPSCs) derived from reprogrammed adult somatic cells have great potential for generating cells for cell replacement and regenerative strategies (Shi et al., 2017; Deinsberger et al., 2020). Transplantation of hiPSC-derived NSPCs improves regeneration in animal models of spinal cord injury (SCI) by replacing lost neurons and providing a permissive substrate that imparts trophic support and promotes long-distance axonal growth (Lu et al., 2014; Lien et al., 2019). Although hNSPCs contribute to neurorepair, changes in the molecular and cellular composition of the host lesion area after traumatic injury impede regenerative efforts (Katoh et al., 2019; Zheng and Tuszynski, 2023). Understanding how traumatic injury and severe changes in the host environment regulate the functionality of hNSPCs will be instrumental for harnessing the potential of these cells to promote CNS repair.

A hallmark of SCI is disruption of the blood–brain barrier and leakage of blood-derived molecules into the parenchyma (Popovich et al., 1996), drastically altering the extracellular environment of cell grafts (Ahuja et al., 2020; Grade et al., 2022). Fibrinogen is a blood-derived factor that is the major architectural protein component of blood clots. Fibrinogen leakage and deposition are general features after SCI and fibrinogen is deposited as early as 1 day after injury, peaks at 7 days, and decreases in the following weeks (Schachtrup et al., 2007). Fibrinogen is a multi-faceted signaling molecule that interacts with integrin and non-integrin receptors and functions as a carrier of growth factors, regulating their bioavailability (Petersen et al., 2018). Recent evidence has expanded the role of fibrinogen beyond blood clotting to orchestration of inflammation and neurorepair (Petersen et al., 2017; Pous et al., 2020; Mendiola et al., 2023). However, the role of fibrinogen in hNSPC differentiation remains unknown.

The maintenance and differentiation of hNSPCs is strongly influenced by the finely tuned host cellular and molecular environment (Katoh et al., 2019; Zheng and Tuszynski, 2023). The lesion microenvironment after SCI hampers the survival of transplanted NSPCs (Tetzlaff et al., 2011; Ahuja et al., 2020) and depending on the cell source induces their differentiation predominantly into astrocytes (Ogawa et al., 2002; O’Shea et al., 2022). After SCI, immune-mediated effects and direct effects of extrinsic factors, including deficiency of neurotrophins and increased deposition of chondroitin sulfate proteoglycans (CSPGs), hinder the functionality of transplanted NSPCs (Donnelly and Popovich, 2008; Karimi-Abdolrezaee et al., 2010; Blesch et al., 2012). Recently, we identified blood-derived fibrinogen as an extrinsic factor that remodels the extracellular matrix and impairs the innate neurogenic repair response of the subventricular zone (SVZ) stem cell niche by inducing NSPC-derived astrogenesis (Bohrer et al., 2015; Pous et al., 2020). Fibrinogen induces activation of the bone morphogenic protein type I receptor (BMPRI) pathway, triggering expression of the transcriptional regulator inhibitor of DNA binding protein 3 (ID3) (Chu et al., 2021) and promoting expression of astrocyte-specific genes (e.g., glial fibrillary acidic protein [*GFAP*] and *GLAST* [also known as *Slc1a3*]) by restraining the transcriptional activity of the basic helix-loop-helix transcription factor E47 (Bohrer et al., 2015; Pous et al., 2020). Given that fibrinogen acts as a provisional extracellular matrix that induces neuroinflammation, alters the composition of the lesion environment, and directly regulates NSPC differentiation, it may regulate the identity of hNSPCs and alter the regeneration processes after SCI. Here, we hypothesize that modifying transplantable hiPSC-derived NSPCs by disrupting ID3 signaling may determine their cell fate and improve their regenerative capacity upon inhibitory fibrinogen impact, offering a novel strategy to enhance their therapeutic potential for SCI repair.

## Methods

### Animals

Adult C57BL/6 female mice aged 8–12 weeks (Jackson Laboratory, Bar Harbor, ME, USA) and weighing between 16–21 g were used. All animals were housed in standard, controlled laboratory conditions (4–5 mice per cage) with a constant temperature of 20–24°C, relative humidity maintained between 45%–65%, and a 12-hour light/12-hour dark cycle. All animal experiments were approved by the Institutional Animal Care and Use Committee of Veterans Affairs (VA) San Diego Healthcare System and were reported according to the Animal Research: Reporting of *In Vivo* Experiments (ARRIVE) guidelines (Percie du Sert et al., 2020).

### Spinal cord contusion injury

The surgical procedure was performed as previously described (Sliwinski et al., 2018). Briefly, mice were deeply anesthetized with intraperitoneal (i.p.) injection of a cocktail (5 mL/kg) containing ketamine (62.5 mg/kg, Serumwerk Bernburg AG, Bernburg, Germany), acepromazine (0.325 mg/kg, Sanofi-Ceva, Düsseldorf, Germany), and xylazine (14.5 mg/kg, Bayer, Leverkusen, Germany). A dorsal laminectomy at vertebral level T9 (corresponding to T11 spinal cord) was performed followed by a moderate contusion with a force of 75 kDyn via the Infinite Horizon Impact Device (IH-0400 Impactor; Precision Systems & Instrumentation, Lexington, KY, USA) equipped with a standard mouse steel-tipped impactor with a diameter of 1.3 mm. Briefly, the spinal processes of the adjacent vertebral bodies rostrally (T8) and caudally (T10) from the site of laminectomy were used to stabilize the spinal column using extra fine forceps attached to the impactor device. The exposed spinal cord was aligned perpendicular to the impactor tip. The position of the impactor tip was adjusted if necessary and positioned 3–4 mm above the exposed spinal cord. After contusion, the paravertebral muscle layers were sutured and the skin incision was stapled. To compensate for dehydration during surgery, animals were given a subcutaneous (s.c.) injection of lactated Ringer’s solution (100 μL) immediately after surgery. Post-surgical care included close observation, manual bladder voiding twice per day, and treatment with antibiotics for about 3 days after surgery. Analgesics (0.01 mg/kg buprenorphine-HCL, 100 μL s.c.) were administered to injured animals immediately after recovery from anesthesia and twice daily during the first 2 post-operative days. Manual bladder evacuation was performed twice a day until reflex bladder function returned.

### Ancrod treatment

To investigate the role of fibrinogen in the formation of an inhibitory environment, C57BL/6 mice were depleted of fibrinogen with ancrod (NIBSC, Hertfordshire, UK) (Akassoglou et al., 2002; Pous et al., 2020). Briefly, mice received 2.4 U ancrod, 2.0 mg per kg body weight, or NaCl + human serum (control), per day via osmotic minipumps (Model: 1002, 0.25 µL/h, Alzet, Campbell, CA, USA) implanted subcutaneously in their back 3 days before SCI. Ancrod was secreted for the duration of the experiment until the mice were perfused.

### Motor recovery assessment

Recovery of motor function was assessed with the Basso Mouse Scale (BMS) for locomotion (Basso et al., 2006). Single mice were placed randomly in an open field and observed by two experienced investigators for at least 5 minutes. The BMS is a 9-point scoring system developed and validated for spinal cord contusion injuries in mice. A score of 0 reflects complete hindlimb paralysis without ankle movement, whereas a score of 9 indicates normal locomotion. Lower scores (0–3) reflect changes in ankle movement and plantar placement of the hind paw; changes in stepping, coordination, and paw position contribute to intermediate scores (4–6); and trunk stability and tail position are assessed for higher scores (7–9). Dorsal and/or plantar stepping starts at a score of 3, a score of 5 indicates frequent or consistent plantar stepping with some coordination, and scores of 6 and 7 indicate mostly coordinated stepping with parallel or rotated paw placement at initial contact and lift off. Motor function was scored on the first day post-injury to confirm that an adequate injury had been induced and then weekly starting 7 days post-injury. Each hind paw was scored individually and then the two scores were averaged to get a single score for each animal per testing day.

### Histology and immunohistochemistry

Mice were transcardially perfused with ice-cold saline, followed by 4% PFA in phosphate buffer under ketamine and xylazine anesthesia, and spinal cord samples were removed, cryoprotected, embedded in Tissue Freezing Medium (Leica Microsystems, Deerfield, IL, USA) and frozen on dry ice. Immunohistochemistry was performed on coronal spinal-cord cryostat sections (25-μm thick), as described previously (Schachtrup et al., 2007, 2015). Briefly, sections were permeabilized in PBS with 0.3% Triton X-100 for 30 minutes, blocked in 5% BSA for 1 hour, and finally incubated overnight with primary antibody in PBS with 0.1% Triton X-100 and 1% BSA. The following primary antibodies were used: mouse anti-CSPGs (1:200, Cat# C8035, Sigma-Aldrich, St. Louis, MO, USA), rat anti-GFAP (1:2000, Cat# 13-0300, Invitrogen, Carlsbad, CA, USA), sheep anti-fibrinogen (1:500, Cat# F4203-02F, US Biological, Swampscott, MA, USA), and goat anti-ionized calcium-binding adapter molecule 1 (IBA-1; 1:500, Cat# ab5076, Abcam, MA, USA), mouse anti-NeuN (1:300, Cat# MAB377, Millipore, Burlington, MA, USA). Secondary antibodies used included donkey antibodies to rabbit, rat, guinea pig, mouse, sheep, and goat conjugated with Alexa Fluor 488, 594, or 405 (1:200, Jackson ImmunoResearch Laboratories, West Grove, PA, USA). Following 1.5 hours of secondary antibody incubation, sections were cover-slipped with 4′,6-diamidino-2-phenylindole (DAPI) Fluoromount-G mounting medium (Southern Biotechnology, Birmingham, AL, USA). For histopathologic analysis of the total spinal cord, the lesion area, and the spared white matter, sections were stained with Luxol fast blue (LFB; Edward Gurr Ltd., London, UK) using standard techniques.

### Caudalized human induced pluripotent stem cell–derived human neural stem/progenitor cell

The hiPSC line used throughout the project was miPS2, which was generated by mRNA reprogramming (Sridhar et al., 2016). Guidelines for clinical-grade hiPSCs and hNSPCs were followed throughout the study (Sullivan et al., 2018).

*hiPSC maintenance:* hiPSCs were cultured as previously described (Sridhar et al., 2016). In short, hiPSCs were maintained in the undifferentiated state with mTeSR1 medium (Stemcell Technologies, Vancouver, Canada) on human embryonic stem-cell-qualified Matrigel (Corning, Steuben County, NY, USA)-coated 6-well plates at 37°C, 5% CO_2_. Passaging of iPSCs was accomplished by enzymatically lifting colonies using ReLeSR (Stemcell Technologies) for approximately 5 minutes. Colonies were then mechanically disrupted to yield small aggregates of cells and split at a 1:6 ratio.

*hiPSC differentiation into hNSPCs:* hiPSCs were differentiated into hNSPCs according to previously described protocols (Krencik and Zhang, 2011). For caudalization, Neural Induction Medium [DMEM-F12, penicillin/streptomycin (1%), MEM NEAA (1%), N2 supplement (1X)] was supplemented with retinoic acids (RA) (0.5 μM, Sigma-Aldrich) from day 10 to day 21. Between days 21 and 90, hNSPCs were maintained in differentiation medium with 20 ng/mL rhFGF2 (Invitrogen) and 20 ng/mL rhEGF (Sigma-Aldrich) and triturated regularly to switch from the neurogenic to the gliogenic phase. Differentiation medium was used throughout this study, consisting of 120 mL of D-MEM/F-12, 120 mL of D-MEM, 2.5 mL of NEAA, 5 mL of B27 without vitamin A, penicillin/streptomycin (1%), and Heparin (2 μg/mL). To enrich the glial progenitor cell fraction, hNSPCs older than 5 months were used throughout the study.

### Generation of modified human induced pluripotent stem cell–derived human neural stem/progenitor cells

Nucleofection and gene editing of hiPSCs: The gene editing of the ID3 locus of hiPSCs was carried out according to previously described protocols(Hammad et al., 2024). Briefly, a total of 4 × 10^5^ hiPSCs were resuspended with Cas9 protein and 100 pmol guide RNA (gRNA) in P3 Nucleofector buffer (Lonza, Rockville, MD, USA). The mixture was transferred to a 16-strip cuvette and electroporated with a 4D Nucleofector X kit (Lonza). Afterward, cells were transferred into pre-warmed complete medium. Untransfected (GFP^–^) and transfected (GFP^+^) cells were harvested 24 hours after the nucleofection to assess transfection efficiency via Accuri C6 flow cytometer (BD Biosciences, San Jose, CA, USA). The following gRNAs were used in this study (5′–3′):

gRNA1: TGG CCA GAC TGC GTT CCG AC

gRNA2: CGA GGC GGT GTG CTG CCT GT

gRNA3: AAG GCG CTG AGC CCG GTG CG

*Gene editing outcome evaluation:* Genomic DNA of transfected cells was extracted with a genomic DNA extraction kit (Macherey Nagel, Düren, Germany). The ID3 target site was amplified using the following primers:

ID3: Fwd 5′-TCC AGG CAG GCT CTA TAA GTG AC -3′

Rev 5′-AGA GGG AAG AGT TAC GCG AGG -3′

The PCR amplicons were purified with a QIAquick PCR Purification Kit (Qiagen, Hilden, Germany). For the T7E1 assay, the DNA fragments were subjected to digestion with the mismatch-sensitive T7 endonuclease I (T7E1, New England BioLabs, Ipswich, MA, USA). DNA was denatured at 95°C for 5 minutes, slowly cooled down to room temperature to allow for the formation of heteroduplex DNA, treated with 5 U of T7E1 for 15 minutes at 37°C, and then analyzed via 2% agarose gel electrophoresis. Quantification of cleavage was performed in ImageJ software (version 1.53a, NIH, Bethesda, MD, USA). All Sanger sequencing was outsourced (Genewiz, Leipzig, Germnay) and coupled with TIDE (Tracking Indels by Decomposition; https://tide.nki.nl) and ICE (Inference of CRISPR Edits; https://www.synthego.com) analyses.

*Pluripotency analysis:* To analyze the pluripotency status of modified hiPSCs, cells were washed and stained in flow cytometry buffer composed of PBS supplemented with 2% FBS at 4°C in the dark for 20–30 minutes before data acquisition on an Accuri C6 flow cytometer. The following antibodies were used: mouse anti-TRA-1-60-PE (1:50, Cat# 12-8863-82, Invitrogen), rabbit anti-OCT-4-AF488 (1:50, Cat# 5177S, Cell Signaling Technology, Danvers, MA, USA), mouse anti-IgM-PE (1:50, Cat# 555584, BD Pharmingen, CA, USA), rabbit anti-IgG-AF488 (1:50, Cat# 4340S, Cell Signaling Technology).

*hiPSC genetic analysis:* To verify genomic stability, genomic DNA of hiPSCs was assessed for recurrent genetic abnormalities with the iCS-digital PSC test, provided as a service by Stem Genomics (https://www.stemgenomics.com), as described previously (Assou et al., 2020).

*Mycroplasma testing:* Mycoplasma contamination was assessed in the conditioned medium of hiPSCs and hiPSC-derived NSPCs by the MycoSPY® Master Mix - PCR Mycoplasma Test Kit (Biontex Laboratories GmbH, Munich, Germany), according to the manufacturer’s instructions. The negative internal control showed a band at 200 bp, while the amplicon of the mycoplasma genome was expected at 500 bp.

### Differentiation of human induced pluripotent stem cell–derived human neural stem/progenitor cells

hNSPCs were plated on poly-D-lysine/laminin-coated eight-well culture slides (Falcon, Corning, NY, USA) at a density of 20,000 cells per 1-cm^2^ growth area in hNSPC culture medium without rhFGF2 or rhEGF. Cells were supplemented with 20 ng/mL CNTF to yield GFAP-positive astrocytes (Krencik and Zhang, 2011). For fibrinogen treatment, hNSPCs were treated with hirudin to prevent fibrinogen cleavage into fibrin (0.5 Units, Sigma-Aldrich) for 15 minutes and then treated with fibrinogen (2.5 mg/mL, Calbiochem, San Diego, CA, USA). For signaling pathway inhibitory assays, cells were pretreated 1 hour before fibrinogen treatment with LDN193189 dihydrochloride (500 nM, Sigma-Aldrich) to block BMP signaling by antagonizing the intracellular kinase domain of BMPRI. hNSPCs were differentiated for 5 days and processed for immunocytochemistry. To ensure equivalent fractions of glia progenitor cells in the wild-type (WT) and genetically edited hNSPCs, hNSPCs of the same culture age were used in each experiment. This procedure was repeated across three independent hNSPC batches. To examine the effect of fibrinogen on neurogenesis, caudalized hNSPCs were generated as described above, but cells were treated with 0.1 µM RA and no rhEGF/rhFGF2 was added. hNSPCs at the early neurogenic stage (day 39; hereafter termed hiPSC-derived neuronal progenitors) were treated with fibrinogen with or without LDN193189 and differentiated for 9 days.

### Immunocytochemistry

Cells were rinsed with ice-cold PBS, fixed in 4% PFA for 30 minutes at 4°C, permeabilized for 10 minutes at 4°C in PBS plus 0.2% Triton X-100 (by volume), blocked in PBS with 5% BSA (Sigma-Aldrich) for 1 hour at room temperature, and incubated with primary antibody at 4°C overnight. The following primary antibodies were used: goat anti-GFAP (1:500, Cat# ab53554, Abcam), rat anti-GFAP (1:1000, Cat# 13-0300, Invitrogen), mouse anti-βIII-tubulin (TUJ-1; 1:100, Cat# MAB1637, Millipore), rat anti-HOXB4 (1:50, RRID: AB_2119288, DSHB, Iowa City, IA, USA), goat anti-human OTX2 (1:150, Cat# AF1979, R&D Systems, Minneapolis, MN, USA), rabbit anti-connexin 43 (1:300, Cat# ab11370, Abcam), rabbit anti-S100ß (1:100, Cat# ab52642, Abcam), rabbit anti-CD44 (1:500, Cat# ab157107, Abcam), and chicken anti- Nestin (NES) (1:500, Cat# CH22123, Neuromics, MN, UsSA). Cells were rinsed and incubated with secondary antibodies conjugated with Alexa Fluor 488, 594, or 647 (1:200, Jackson ImmunoResearch Laboratories) for 1.5 hours in the dark at room temperature. After washing in PBS, cells were cover-slipped with DAPI Fluoromount-G mounting medium (Southern Biotechnology, AL, USA).

### Quantitative real-time PCR

RNA was isolated from hiPSCs and/or hNSPCs with an RNeasy Kit (Qiagen) and cDNA was reverse transcribed using the High-Capacity cDNA Reverse Transcription Kit (Applied Biosystems^TM^, MA, USA) according to the manufacturer’s instructions. Quantitative real-time PCR was performed as described (Bohrer et al., 2015) with CFX Connect Real-Time PCR Detection System (Bio-Rad, CA, USA) and the relative mRNA expression level was normalized to GAPDH. The following primers were used:

GAPDH: Fwd 5′-TGC ACC ACC AAC TGC TTA GC -3′

Rev 5′-GGC ATG GAC TGT GGT CAT GAG -3′

ID3: Fwd 5′-GAG AGG CAC TCA GCT TAG CC -3′

Rev 5′-TCC TTT TGT CGT TGG AGA TGA C -3′

### Immunoblots

For the detection of BMP-2-induced ID3 expression in hiPSCs, hiPSCs were treated with BMP-2 (2 ng/mL, Peprotech). For the detection of fibrinogen-induced ID3 expression, hNSPCs were treated with fibrinogen or left untreated for various durations. Protein concentrations were determined by Bradford assay (Bio-Rad, CA, USA) and equal amounts of protein (20 µg per lane) were separated by SDS-PAGE and subsequently transferred onto polyvinylidene difluoride (PVDF) membranes. Blots were blocked with 5% nonfat milk in TBS-T at room temperature for 1 hour, and incubated with primary antibody at 4°C overnight. The following primary antibodies were used: rabbit anti-AQP4 (1:300, Cat# sc-20812, Santa Cruz Biotechnology, TX, USA), rat anti-GFAP (1:2000, Cat# 13-0300, Invitrogen), mouse anti-β-actin (1:1000, Cat# A1978, Sigma-Aldrich), rabbit anti-ID3 (1:2000, Cat# M101, CalBioreagents, CA, USA) and mouse anti-α-tubulin (1:1000, Cat# T6199, Sigma-Aldrich). Blots were washed three times with TBS-T, incubated with peroxidase-labeled secondary antibodies (1:5000, Cell Signaling Technology) diluted in 5% nonfat milk in TBS-T for 1 hour at room temperature, and washed again, followed by detection with chemiluminescence (ECL, GE Healthcare, MA, USA). Western blots were quantified in ImageJ (version 1.53a, NIH, USA). Densitometry was performed with values for each band normalized to the corresponding α-tubulin loading controls from the same membrane.

### Isolation and characterization of extracellular vesicles

Isolation and characterization of extracellular vesicles (EVs) were performed according to the MISEV2018 guidelines (Thery et al., 2018). Secretory EVs in culture media were purified via differential centrifugations(Morton et al., 2019). Briefly, **~**3 × 10^6^ hNSPCs were cultured for 24 hours in EV-free medium, which was prepared by subjecting differentiation media as mentioned above to ultracentrifugation at 100,000 × *g* for 90 minutes at 4°C to deplete vesicular particles. The conditioned medium was collected and underwent differential centrifugation at 4 °C as follows: 300 g for 5 minutes, 2000 g for 10 minutes, 100 000 g for 90 minutes (45 Ti rotor and Optima L-80 ultracentrifuge, Beckman Coulter, CA, USA), then pellet was washed with PBS and centrifuged at 100 000 g for 90 minutes, twice. The particle size of EV samples was measured by nanoparticle tracking analysis (NTA) with a ZetaView NTA instrument equipped with a 488-nm laser (Particle Metrix GmbH, Inning am Ammersee, Germany). The size distribution was measured in scatter mode and measurements were obtained at 11 different positions. Image evaluation was done on particles with the following parameters: minimum brightness: 30; minimum area: 10; maximum area: 1000. The particles from all recorded videos were collected in a single table and then the joint particle-size distribution (PSD) histogram, mean size, and total particle concentration, corrected by the dilution factor used for each particular sample, were calculated. The morphology of the EVs was verified by transmission electron microscopy (TEM) (Zeiss LEO906, Carl Zeiss Microscopy GmbH, Oberkochen, Germany). Briefly, after 5 minutes of adsorption, EV pellets were fixed in 1% glutaraldehyde for 5 minutes. The EV pellets were then rinsed four times in ddH_2_O, and negative staining was carried out for 1 minute with 1% uranyl acetate. Negative staining is a method in which contrast is not applied to the object but to its environment, with the result resembling an inverted traditional TEM image. EVs were then digitally photographed with an inverted traditional TEM. The final EV concentration was quantified with a BCA Protein Assay Kit (Thermo Fisher Scientific, MA, USA). EV-associated markers were detected by immunoblots with the procedure that proteins were first separated using 8%–16% nUView Tris-Glycine Precast Gels (Biotrend, Cologne, Germany) and subsequently transferred onto polyvinylidene difluoride (PVDF) membranes. Blots were blocked with 5% nonfat milk in TBS-T at room temperature for 1 hour, and incubated with primary antibody at 4°C overnight. The following primary antibodies were used: EV markers: rabbit anti-CD63 (1:1000, Cat# 25682-1-AP, Proteintech, IL, USA), rabbit anti-CD9 (1:1000, Cat# ab236630, Abcam), rabbit anti-CD81 (1:1000, Cat# 56039S, Cell Signaling Technology), mouse anti-ALIX (1:1000, Cat# 2171, Cell Signaling Technology); cellular marker: mouse anti-β-actin (1:1000, Cat# A1978, Sigma-Aldrich); negative control: rabbit anti-APOB (1:1000, Cat# ab139401, Abcam). Blots were washed three times with TBS-T, incubated with peroxidase-labeled secondary antibodies (1:5000, Cell Signaling Technology) diluted in 5% nonfat milk in TBS-T for 1.5 hours at room temperature, and washed again, followed by detection with chemiluminescence (ECL, GE Healthcare, MA, USA). Total protein on the stain-free gel was imaged with UVP GelStudio equipment (Analytik Jena, Jena, Germany). The immunoblots were quantified in ImageJ. Densitometry was performed with values for each band normalized to the corresponding total protein loading.

### Neurite outgrowth assays

Mouse cerebellar granule neurons (CGNs) were isolated as described previously (Schachtrup et al., 2007). Mouse CGNs were plated into poly-D-lysine-coated eight-well culture slides (BD Falcon) at a density of 7.5 × 10^4^/well and treated for 24 hours with 100 µg (approximately 4 × 10^9^) EV particles isolated from either WT or *ID3*^–/–^ hNSPCs. For fibrinogen treatment, mouse CGNs were first treated with hirudin (0.5 units, Sigma-Aldrich) for 15 minutes, followed by fibrinogen (2.5 mg/mL, Calbiochem) with or without 100 µg (approximately 4 × 10^9^) EV particles isolated from either WT or *ID3*^–/–^ hNSPCs. Neurons extended processes by 24 hours and were then fixed in 4% paraformaldehyde and stained with rabbit anti-β-tubulin-III (1:1000, Cat# ab18207, Abcam) as described in the section of “Immunocytochemistry”. Quantification of neurite length and percent of cells with neurites was performed in ImageJ as described previously (Schachtrup et al., 2007).

### Small-RNA-sequencing analysis of extracellular vesicles

The total RNA in EVs was isolated with a mirVana miRNA Isolation Kit (Invitrogen) according to the manufacturer’s protocol, and measured with a Qubit RNA Assay (Thermo Fisher Scientific). Small RNA sequencing and transcriptome data analysis were performed by Novogene (Beijing, China). In brief, the exosome small RNA library was generated using a SMART-Seq v4 Ultra-low Input RNA Kit (TakaraBio, Japan), and single-end 50-bp sequencing was performed on an Illumina platform at a depth of 10 million reads per sample. Raw data (raw reads) in the fastq format were first processed with in-house perl scripts. Clean data (clean reads) were obtained by removing reads containing adapters, poly-N, and low-quality nucleotides. Small RNA reads were mapped to the genome with Bowtie. Differential expression analysis was performed by DESeq2 (Love et al., 2014). The *P* values were adjusted via the Benjamini-Hochberg method. Genes with adjusted *P* value < 0.05 and |log2 (FoldChange)| > 1 were considered to be differentially expressed. Gene Ontology (GO) and Kyoto Encyclopedia of Genes and Genomes (KEGG) pathway enrichment analysis of differentially expressed genes was implemented via the clusterProfiler R package (Yu et al., 2012) with a corrected *P* value < 0.05. The miRNA target-based network was constructed using Cytoscape (v3.10.3) (Shannon et al., 2003). Within the constructed network, core miRNAs were identified using cytoHubba (Chin et al., 2014), a plugin in Cytoscape, with Maximal Clique Centrality (MCC) metric.

### Confocal microscopy and imaging analysis

For mouse tissue, Z-stack images (1 µm per stack) were acquired by a Leica TCS SP8 laser confocal microscope (Leica Microsystems, Wetzlar, Germany) with 20× and 40× oil-immersion objectives. Representative images were acquired by combining Z-stack images into a maximum intensity projection with the LAS AF image analysis software (Leica Microsystems, Wetzlar, Germany). For LFB and immunocytochemistry staining, images were acquired with an Axio Imager M2 epifluorescence microscope equipped with dry plan-apochromat objectives (10×/0.45 NA; 20×/0.80 NA) and the ZEN image analysis software (Carl Zeiss Microscopy GmbH, Oberkochen, Germany).

Fibrinogen immunoreactivity analysis: The quantified lesion areas were selected based on the DAPI^+^ and GFAP^+^ cell layer defining the lesion rim. Imaging was performed on the lesioned area of the spinal cord. Six randomly sampled sections were imaged per mouse. The maximal projected signal was subjected to threshold processing and the immunoreactivity was analyzed in ImageJ software with the “Area integrated density measurement” tool. Total IR was calculated as the percentage area density, defined as the number of pixels (positively stained areas) divided by the total number of pixels (sum of positively and negatively stained areas) in the imaged field.

GFAP, CSPG, and CD68 immunoreactivity analysis: A rectangular area 100 µm × 300 µm was delineated for quantification purposes. Three such areas were acquired for each of the following regions within the spinal-cord section: lesion core, lesion rim, and penumbra. The selection of the quantified lesion areas was guided by the presence of DAPI^+^ and GFAP^+^ cell layers, which defined the lesion rim. Interior to the lesion rim corresponds to the lesion core, while the area outside the lesion rim represents the penumbra (Conforti et al., 2022). Six randomly sampled sections were imaged per mouse. The maximal projected signal was subjected to threshold processing and the immunoreactivity was analyzed in ImageJ with the “Area integrated density measurement” tool. Total IR was calculated as the percentage area density, defined as the number of pixels (positively stained areas) divided by the total number of pixels (sum of positively and negatively stained area) in the imaged field.

NeuN^+^ neuron analysis: For quantification of NeuN^+^ neurons in the spinal cord grey matter, ±2000 µm to the injury epicenter at 1000-μm intervals in caudal directions, were analyzed in ZEN 3.0 blue edition (Carl Zeiss Microscopy GmbH, Oberkochen, Germany). Six randomly sampled sections were imaged per mouse. For each section, the area was measured with the ZEN “area measurement” tool, while the NeuN^+^ cell count was measured by ImageJ. Lesion size analysis: For quantification of the total cord and white matter area, LFB-stained sections, ±2000 µm to the injury epicenter at 1000-μm intervals, were analyzed in ZEN 3.0 blue edition. White matter was identified by the blue LFB staining. The injury epicenter was defined as the section with the largest cross-sectional area of lesioned tissue, identified by dense fibrous tissue with an inconsistent matrix. For each section, the area was measured with the ZEN “area measurement” tool.

### Statistical analysis

Data are shown as the means ± SEM. Differences between groups were examined by one-way ANOVAs for multiple comparisons, followed by Bonferroni’s correction for comparisons of means. One-tailed Student’s *t*-tests were used to analyze differences between pairs of experimental groups. Analyses were conducted using GraphPad Prism (GraphPad Software, v6.0, La Jolla, USA). Differences were considered significant when the *P* value was < 0.05.

## Results

### Fibrinogen induces human induced pluripotent stem cell–derived neural stem/progenitor cell differentiation into astrocytes

**Figure 1:**
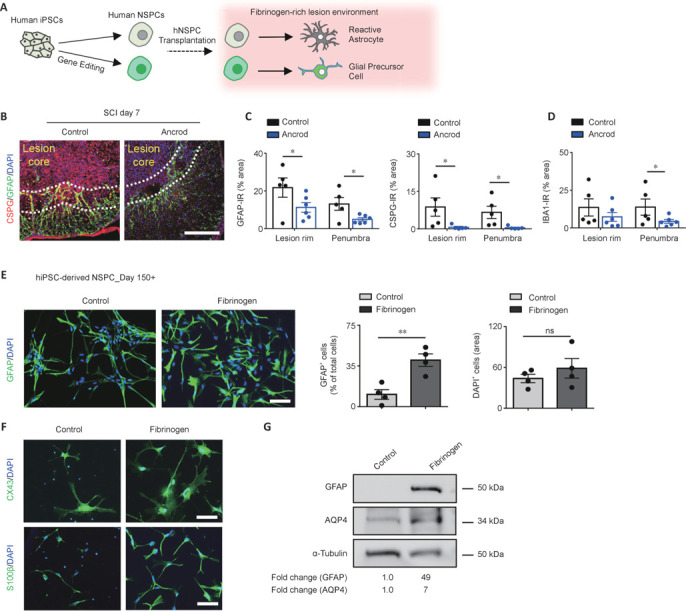
Fibrinogen induces hiPSC-derived NSPC differentiation into astrocytes. (A) Schematic of the potential beneficial effects of modified hNSPCs after SCI. (B) Immunolabeling for CSPG (red) and GFAP (green) in the spinal cord of fibrinogen-depleted mice (ancrod) and control animals (NaCl) 7 days after SCI (lesion rim area is indicated by dashed lines). (C) Quantification of GFAP (left) and CSPG (right) immunoreactivity (IR) for the lesion rim and penumbra in the spinal cord of fibrinogen-depleted mice and control mice (ancrod, *n* = 6 mice; NaCl, *n* = 5 mice) 7 days after SCI. (D) Quantification of IBA-1 immunoreactivity (IR) for the lesion rim and penumbra in the spinal cord of fibrinogen-depleted mice and control mice (ancrod, *n* = 6 mice; NaCl, *n* = 5 mice) 7 days after SCI. (E) Immunolabeling of GFAP^+^ astrocytes (green) 5 days after fibrinogen treatment of hNSPCs or no treatment (control). Quantification of GFAP^+^ cells and DAPI^+^ cells (*n* = 4). (F) Representative images of Connexin 43^+^ (green, top) and S100β^+^ (green, bottom) astrocytes 5 days after fibrinogen treatment (*n* = 2). (G) Immunoblot for GFAP and AQP4 in hNSPCs 2 days after fibrinogen treatment (*n* = 2). Scale bars: 200 µm (B), 100 µm (E, F). Data are presented as the mean ± SEM. Unpaired Student’s *t*-tests (C–E) were performed. *P < 0.05, **P < 0.01. AQP4: Aquaporin-4; CSPG: chondroitin sulfate proteoglycan; DAPI: 4′,6-diamidino-2-phenylindole; hiPSC: human induced pluripotent stem cell; hNSPC: human neural stem/progenitor cell; ns: not significant; NSPC: neural stem/progenitor cell; SCI: spinal cord injury.

NSPC maintenance and differentiation are strongly regulated by a complex and dynamic extracellular microenvironment (Petersen et al., 2017; Pous et al., 2020). The blood-derived factor fibrinogen induces neurotoxic microglial gene programs in neurodegeneration (Mendiola et al., 2023), activates astrocytes (Schachtrup et al., 2010; Conforti et al., 2022), and alters endogenous SVZ NSPC identity (Pous et al., 2020). Fibrinogen is massively deposited in the lesioned area after SCI (Schachtrup et al., 2007) and thus might alter the composition of the lesion environment to regulate the identity of transplanted hNSPCs, impairing the regeneration process (**Figure 1A**). Measured at 7 days post-injury, acute fibrinogen depletion by the pharmacologic reagent ancrod (Pous et al., 2020; **Additional Figure 1A**) resulted in a **~**40% and **~**60% reduction in GFAP immunoreactivity (IR) in the lesion rim and penumbra, respectively, compared with control mice, and in a **~**90% reduction in inhibitory CSPGs in both the lesion rim and the penumbra (**Figure 1B** and **C**). Furthermore, fibrinogen depletion by ancrod resulted in a **~**70% reduction of IBA-1 IR in the penumbra at 7 days post-injury compared with control mice (**Figure 1D** and **Additional Figure 1B**). Fibrinogen depletion by ancrod did not affect the NeuN^+^ neurons, lesion size, or behavioral outcomes (**Additional Figure 1C–E**). These data suggest that fibrinogen is massively deposited after SCI and induces inflammation, astrocyte activation, and the deposition of inhibitory extracellular factors.

Because fibrinogen is massively deposited in the spinal cord parenchyma after traumatic injury, we next investigated the direct effect of fibrinogen on transplantable hNSPCs. We initially established long-term cultures of region-specific hNSPCs exhibiting a spinal cord phenotype (**Additional Figure 2**). These caudalized hNSPCs retain multipotency *in vitro*, possessing the intrinsic capacity to differentiate into neurons, astrocytes, and oligodendrocytes, while prolonged culture conditions lead to an enrichment of the glial progenitor cell population (Krencik and Zhang, 2011). Notably, fibrinogen robustly induced hNSPC differentiation into astrocytes, revealed by increased abundance of GFAP^+^, Connexin 43^+^, and S100β^+^ cells (**Figure 1E** and **F**) and an increase in GFAP and AQP4 protein expression (**Figure 1G**). Overall, these data suggest that fibrinogen potently induces hNSPC differentiation into astrocytes.

### Fibrinogen induces differentiation of human induced pluripotent stem cell–derived neural stem/progenitor cells into astrocytes via the BMPRI–inhibitor of DNA binding 3 axis

**Figure 2:**
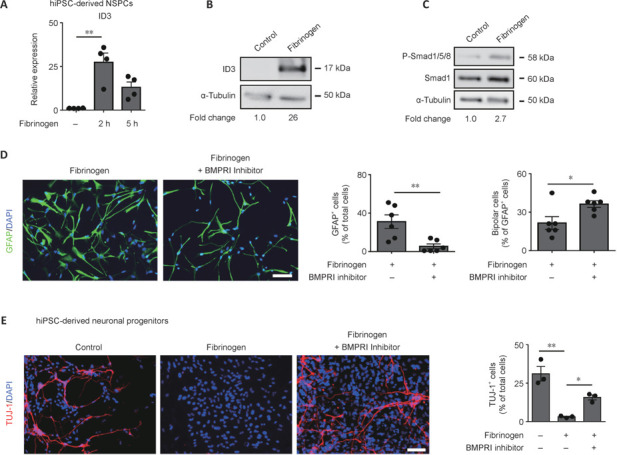
Fibrinogen induces hiPSC-derived NSPC differentiation into astrocytes via the BMPR–ID3 axis. (A) Relative expression of *ID3* mRNA in hNSPCs, untreated or 2 and 5 hours after fibrinogen treatment, determined by quantitative PCR (*n* = 4). (B) Immunoblot for ID3 in hNSPCs 1 hour after fibrinogen treatment (*n* = 2). (C) Immunoblot for P-Smad1/5/8 and Smad1 in hNSPCs 1 hour after fibrinogen treatment (*n* = 2). (D) Immunolabeling of GFAP^+^ astrocytes (green) in hNSPCs pretreated with a BMP receptor inhibitor 1 hour before fibrinogen treatment, 5 days after initiation of differentiation. Quantification of GFAP^+^ astrocytes (left) and bipolar cells (right) (*n* = 6, a minimum of 110 GFAP^+^ cells were analyzed per condition). (E) Immunolabeling of TUJ-1^+^ neurons (red) in hiPSC-derived neuronal progenitors pretreated with a BMP receptor inhibitor 1 hour before fibrinogen treatment, 5 days after initiation of differentiation. Quantification of TUJ-1^+^ neurons in hiPSC-derived neuronal progenitors (*n* = 3). Scale bars: 100 µm (D, E). Data are expressed as the mean ± SEM. Unpaired Student’s *t*-tests (D) and a one-way analysis of variance with Bonferroni’s multiple comparisons (A, E) were performed. *P < 0.05, **P < 0.01. BMPR: BMP receptor; DAPI: 4′,6-diamidino-2-phenylindole; GFAP: glial fibrillary acidic protein; hiPSC: human induced pluripotent stem cell; ID: inhibitor of DNA binding; hNSPC: human neural stem/progenitor cell; NSPC: neural stem/progenitor cell; TUJ-1: β-tubulin.

Next, we analyzed the molecular mechanisms that fibrinogen utilizes to induce differentiation of hNSPCs into astrocytes. Upon fibrinogen treatment, hNSPCs showed increased ID3 expression and phosphorylation of the BMP signal transducers Smad1/5/8 (**Figure 2A–C**), consistent with our previous findings in rodent SVZ NSPCs (Pous et al., 2020). The selective inhibitor of BMP type I receptor kinases, LDN-193189 (Cuny et al., 2008), significantly reduced the fibrinogen-mediated differentiation of adult hNSPCs into astrocytes and increased the proportion of GFAP^+^ cells with a bipolar morphology (**Figure 2D**), indicating that fibrinogen-triggered activation of the BMP type I receptor pathway is necessary for the induction of hNSPC differentiation into astrocytes. Moreover, pre-treatment of hiPSC-derived neuronal progenitor cells with the BMPRI inhibitor significantly attenuated the inhibitory effect of fibrinogen on TUJ-1^+^ cells (**Figure 2E**). Together, these data suggest that fibrinogen deposition in the spinal cord parenchyma after injury rapidly upregulates ID3, which in turn promotes hNSPC differentiation into astrocytes.

### Generation of inhibitor of DNA binding 3-depleted human induced pluripotent stem cell–derived neural stem/progenitor cells via CRISPR/Cas9

**Figure 3:**
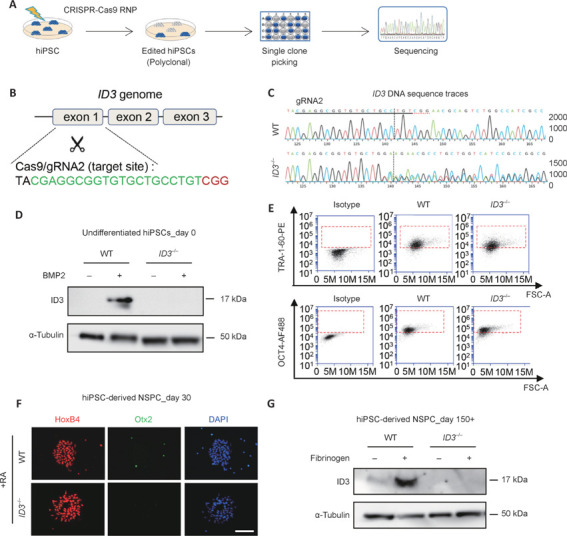
Generation of ID3-depleted hiPSC-derived NSPCs via CRISPR/Cas9. (A) Scheme illustrating the process of the generation of *ID3*^–/–^ hiPSCs via the CRISPR/Cas9 ribonucleoprotein (RNP) system. (B) Schematic of the gene-targeting strategy. The gRNAs were designed to target the first exon of the ID3 locus. The target site sequence of gRNA2 is shown. (C) Sanger sequence view showing WT and *ID3*^–/–^ hiPSC genome sequences in the region around the target sequence. The horizontal black underline represents the gRNA2 target site. The horizontal red dotted underline represents the protospacer adjacent motif (PAM) site. The vertical black dotted line indicates the actual CRISPR/Cas9 cut site. (D) Immunoblot for ID3 in WT and *ID3*^–/–^ undifferentiated hiPSCs 1 hour after BMP2 treatment (*n* = 3). (E) Flow cytometry analysis of the pluripotency markers TRA-1-60 and OCT4 in WT and *ID3*^–/–^ hiPSCs. WT hiPSCs stained with an isotype control antibody were used as a negative control. (F) Immunolabeling for the caudal marker HOXB4 (red), and rostral brain marker OTX2 (green) in retinoid acid (RA)-treated WT and *ID3*^–/–^ hNSPCs (similar data were obtained for multiple batches, *n* = 3). (G) Immunoblot for ID3 in caudalized WT and *ID3*^–/–^ hNSPCs 1 hour after fibrinogen treatment (*n* = 2). Scale bar: 70 µm (F). hiPSC: Human induced pluripotent stem cell; hNSPC: human neural stem/progenitor cell; HOXB4: homeobox B4; ID: inhibitor of DNA binding; NSPC: neural stem/progenitor cell; OTX2: orthodenticle homeobox 2; WT: wild-type.

Next, to generate transplantable hNSPCs that are resistant to fibrinogen-induced differentiation into inhibitory astrocytes, we homozygously disrupted the *ID3* locus in hiPSCs (hereafter termed *ID3*^–/–^) using an optimized DNA-free CRISPR/Cas9 ribonucleoprotein (RNP) delivery system that combines high efficiency with minimal off-target activity (Liu et al., 2015; Hammad et al., 2024; **Figure 3A**). We designed three guide RNAs (gRNAs) to target exon 1 of the *ID3* locus (**Figure 3B**), and we assessed the on-target mutagenesis frequency induced by each Cas9/gRNA-RNPs in the hiPSCs with the T7 endonuclease 1 mismatch cleavage (T7E1) assay (**Additional Figure 3A**). Sanger sequencing demonstrated that the Cas9/gRNA2-edited hiPSC polyclonal population, which had the highest activity in the T7E1 assay, resulted in 91% of edited *ID3* alleles (**Additional Figure 3B**). Monoclonal *ID3*-edited hiPSCs were recovered after limiting dilution (**Additional Figure 3C**) and Sanger sequencing revealed successful generation of a homozygous *ID3*^–/–^ hiPSC clone with a 4 bp-deletion on one *ID3* allele and a 7 bp-deletion on the second ID3 allele (**Figure 3C** and **Additional Figure 3D**). Further characterization of *ID3*^–/–^ hiPSCs confirmed the absence of functional ID3 protein upon BMP2 stimulation (**Figure 3D**), while the pluripotency of *ID3*^–/–^ hiPSCs was confirmed by OCT4 and TRA-1-60 expression (**Figure 3E**), and there were no unintended genomic alterations (**Additional Figure 3E** and **F**). Successfully generated *ID3*^–/–^ hiPSCs were induced to differentiate into caudalized hNSPCs (hereafter termed *ID3*^–/–^ hNSPCs), which displayed a HOXB4^+^ distinct caudalized phenotype (**Figure 3F**). Importantly, immunoblotting for fibrinogen-induced ID3 expression demonstrated the successful generation of mature *ID3*^–/–^ hNSPCs (**Figure 3G**).

### Inhibitor of DNA binding 3-depleted human induced pluripotent stem cell–derived neural stem/progenitor cells are resistant to fibrinogen-induced astrogenesis

**Figure 4:**
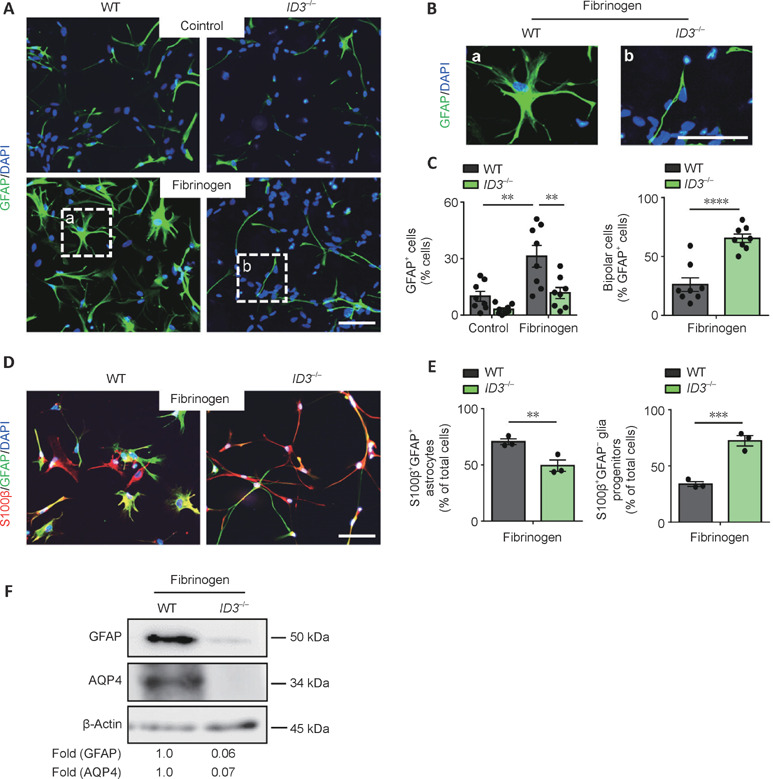
ID3-depleted hiPSC-derived NSPCs are resistant to fibrinogen-induced astrogenesis. (A) Immunolabeling of GFAP^+^ astrocytes (green) in WT and *ID3*^–/–^ hNSPCs 5 days after fibrinogen treatment (bottom) or left untreated (top, control). (B) Dashed boxes in A indicate magnifications of GFAP^+^ astrocytes (green) in WT and *ID3*^–/–^ hNSPCs 5 days after fibrinogen treatment. (C) Quantification of total GFAP^+^ astrocytes (left) and bipolar cells (right) in WT and *ID3*^–/–^ hNSPCs after fibrinogen treatment (*n* = 8, a minimum of 350 GFAP^+^ cells were analyzed per condition). (D) Immunolabeling of S100β (red) and GFAP (green) in differentiated WT and *ID3*^–/–^ hNSPCs 5 days after fibrinogen treatment. (E) Quantification of total S100β^+^GFAP^+^ mature astrocytes (left) and total S100β^+^GFAP^−^ glial progenitor cells (right) derived from WT and *ID3*^–/–^ hNSPCs 5 days after fibrinogen treatment (*n* = 3). (F) Immunoblot for GFAP and AQP4 in WT and *ID3*^–/–^ hNSPCs 2 days after fibrinogen treatment (*n* = 2). Scale bars: 200 µm (A, D), 100 µm (B). Data are expressed as the mean ± SEM. A one-way analysis of variance with Bonferroni’s multiple comparisons (C, left) and unpaired Student’s *t*-tests (C, right, E) were performed. **P < 0.01, ***P < 0.001, ****P < 0.0001. AQP4: Aquaporin-4; DAPI: 4′,6-diamidino-2-phenylindole; GFAP: glial fibrillary acidic protein; hiPSC: human induced pluripotent stem cell; ID: inhibitor of DNA binding; NSPC: neural stem/progenitor cell; WT: wild-type.

To evaluate the regenerative capacity of *ID3*^–/–^ hNSPCs in an astrogenic lesion microenvironment after SCI, we first treated *ID3*^–/–^ hNSPCs with fibrinogen and analyzed their differentiation potential *in vitro*. Remarkably, while fibrinogen induced WT hNSPC differentiation into astrocytes, *ID3*^–/–^ hNSPCs showed significantly reduced differentiation toward GFAP^+^ astrocytes upon fibrinogen treatment, with an increased proportion of bipolar elongated GFAP^+^ cells reminiscent of immature glial progenitor cells (**Figure 4A–C**). Of note, untreated *ID3*^–/–^ hNSPCs differentiated less into GFAP^+^ astrocytes (**Figure 4A** and **C**), indicating a reduced astrogenic potential. Next, we examined the expression of the astrocyte marker GFAP in combination with S100β, a marker for mature astrocytes that have lost their neural stem cell potential (Raponi et al., 2007). *ID3*^–/–^ hNSPCs had a decreased proportion of S100β^+^GFAP^+^ mature astrocytes after fibrinogen treatment but an increased proportion of S100β^+^GFAP^–^ astrocyte-restricted progenitors (**Figure 4D** and **E**). Human glial progenitor cells are characterized by their unique expression of the transmembrane glycoprotein CD44 and S100β, while lacking GFAP expression (Liu et al., 2004; Zhang et al., 2016; Martins-Macedo et al., 2021). Indeed, the *ID3*^–/–^ hNSPC glial progenitor identity upon fibrinogen treatment was further confirmed by their expression of CD44 and Nestin (**Additional Figure 4A**). In line, immunoblotting for GFAP and AQP4, using lysate of *ID3*^–/–^ hNSPCs after fibrinogen treatment, revealed their resistance to astrogenic stimuli (**Figure 4F**). Finally, we observed a notable increase in the differentiation of *ID3*^–/–^ hNSPCs into TUJ-1^+^ cells upon fibrinogen treatment (**Additional Figure 4B**), indicating that *ID3*^–/–^ hNSPCs had an altered glial-progenitor-cell–like morphology and that *ID3*^–/–^ hNSPCs are resistant to astrogenic fibrinogen stimuli.

### Inhibitor of DNA binding 3-depleted human induced pluripotent stem cell–derived neural stem/progenitor cells secrete beneficial extracellular vesicles that support neurite outgrowth

**Figure 5:**
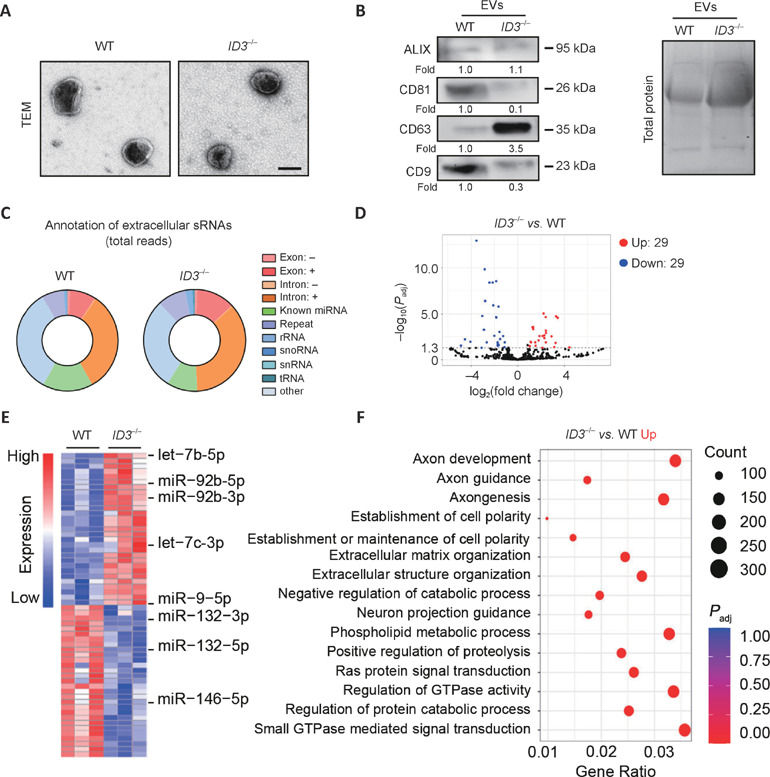
ID3-depleted hiPSC-derived NSPCs secrete EVs with a unique profile of beneficial miRNAs. (A) Representative transmission electron microscopy (TEM) images of EVs secreted by WT and *ID3*^–/–^ hNSPCs, obtained from medium conditioned for 24 hours (*n* = 3). (B) Immunoblot for EV markers (CD9, CD63, CD81, and ALIX) and luminal protein (β-actin) for EVs secreted by WT and *ID3*^–/–^ hNSPCs, obtained from medium conditioned for 24 hours (left). Total protein was detected on a stain-free gel (right) (*n* = 3–8). (C) Annotation of extracellular sRNAs secreted by WT and *ID3*^–/–^ hNSPCs, obtained from medium conditioned for 24 hours (*n* = 3). ‘’exon: −”: exon antisense strand; ‘’exon: +’’: exon sense strand; ‘’intro: –’’: intron antisense strand; ‘’intron: +’’: intron sense strand; Known miRNA: known microRNA; Repeat: repeat sequence; rRNA: ribosomal RNA; snoRNA: small nucleolar RNA; snRNA: small nuclear RNA; tRNA: transfer RNA. (D) Volcano plot showing the overall expression distribution of different miRNAs in EVs secreted by WT and *ID3*^–/–^ hNSPCs, obtained from medium conditioned for 24 hours (*n* = 3). The threshold was set to *P*_adj_ < 0.05. The x-axis shows the fold change in miRNA expression between the different samples, and the y-axis shows the statistical significance of the differences. Statistically significant differences are represented by red (upregulated) and green (downregulated) dots. (E) Hierarchical clustering of the differentially expressed miRNAs in EVs secreted by WT and *ID3*^–/–^ hiPSC-derived NSPCs, obtained from medium conditioned for 24 hours (*n* = 3). (F) The top 15 Gene Ontology (GO) Biological Processes targeted by miRNAs differentially upregulated in EVs secreted by *ID3*^–/–^ hNSPCs compared to WT hNSPCs (*n* = 3). Scale bar: 0.1 µm in A. EV: Extracellular vesicle; hiPSC: human induced pluripotent stem cell; hNSPC: human neural stem/progenitor cell; ID: inhibitor of DNA binding; NSPC: neural stem/progenitor cell; WT: wild-type.

So far, we have shown that depletion of *ID3* in hNSPCs results in morphological differences and a resistance to fibrinogen-induced astrogenesis. To further explore the functionality of *ID3*^–/–^ hNSPCs, we analyzed their extracellular vesicles (EVs), which mediate intercellular communication (Budnik et al., 2016; Losurdo and Grilli, 2020; van Niel et al., 2022) and immune modulation (Morton et al., 2018; Bian et al., 2020; Peruzzotti-Jametti et al., 2021) and thus affect neurorepair. EV isolation procedures and validation were carried out according to the MISEV2018 guidelines (Thery et al., 2018; **Additional Figure 5A**). Transmission electron microscopy (TEM), immunoblotting, and nanoparticle tracking analysis (NTA) revealed that EVs secreted and isolated from WT and *ID3*^–/–^ hNSPCs exhibited typical morphology, size, and concentration (**Figure 5A** and **B**, **Additional Figure 5B–D**). Recent evidence suggests that different levels of tetraspanins, such as CD9 and CD63, may indicate different EV subpopulations as the protein composition of exosomes and small ectosomes differs (Mathieu et al., 2021). Interestingly, immunoblotting showed significant upregulation of CD63 and downregulation of CD9 and CD81 in EVs from *ID3*^–/–^ hNSPCs, suggesting that the composition of EV subtypes in hNSPCs is altered by *ID3* depletion (**Figure 5B**).

Importantly, although EVs from WT and *ID3*^–/–^ hNSPCs contained similar fractions of miRNA (10%–16%), tRNA (0.1%–0.3%), rRNA (1%–2%), and the other ncRNAs (30%–35%) (**Figure 5C** and **Additional Table 1**), small RNA sequencing (sRNA-seq) revealed 58 differentially expressed miRNAs in EVs derived from *ID3*^–/–^ hNSPCs, including 29 miRNAs that were upregulated and 29 that were downregulated compared with WT hNSPCs (**Figure 5D** and **E**, **Additional Table 2**). Among these, several miRNAs, such as miR-9-5p, miR-92b, miR-132, miR-146-5p, let-7b-5p, and let-7c-3p, are known to regulate cell-cycle dynamics, neurodevelopment, and immunomodulation (Willis et al., 2020; Bonetto and Grilli, 2023). Gene ontology (GO) enrichment analysis of all potential target genes showed an upregulation of miRNA targeting genes involved in “neuron projection guidance,” “axon guidance,” “axon development,” “axonogenesis,” and “extracellular matrix organization” in *ID3*^–/–^ hNSPCs (**Figure 5F**). These results highlight that EVs secreted by *ID3*^–/–^ hNSPCs are enriched with miRNAs that promote neurogenesis and neurorepair.

**Additional Table 1:** Annotation of extracellular small RNAs in EVs derived from *ID3*^-/-^ hNSPCs and WT hNSPCs

Types	KO1	KO1 (percent)	KO2	KO2 (percent)	KO3	KO3 (percent)	WT1	WT1 (percent)	WT2	WT2 (percent)	WT3	WT3 (percent)
Total	11125710	100.00%	8475344	100.00%	11751508	100.00%	9454435	100.00%	10998934	100.00%	12387429	100.00%
known_miRNA	943271	8.48%	1298795	15.32%	601744	5.12%	1609677	17.03%	2383935	21.67%	1187390	9.59%
rRNA	204170	1.84%	220872	2.61%	259576	2.21%	107859	1.14%	99591	0.91%	108048	0.87%
tRNA	31760	0.29%	35247	0.42%	50109	0.43%	13765	0.15%	11434	0.10%	11090	0.09%
snRNA	11245	0.10%	4272	0.05%	45743	0.39%	6821	0.07%	7532	0.07%	6610	0.05%
snoRNA	16966	0.15%	11050	0.13%	28859	0.25%	9419	0.10%	11433	0.10%	10715	0.09%
repeat	1317240	11.84%	866615	10.23%	609105	5.18%	634166	6.71%	725495	6.60%	951821	7.68%
novel_miRNA	56	0.00%	24	0.00%	65	0.00%	44	0.00%	73	0.00%	16	0.00%
exon:+	1945402	17.49%	991472	11.70%	884457	7.53%	765731	8.10%	780094	7.09%	1200485	9.69%
exon:-	56805	0.51%	44079	0.52%	174656	1.49%	103260	1.09%	99812	0.91%	90523	0.73%
intron:+	3208074	28.83%	2229143	26.30%	6178914	52.58%	3350947	35.44%	3005049	27.32%	4237994	34.21%
intron:-	40455	0.36%	22384	0.26%	28930	0.25%	26471	0.28%	34447	0.31%	51330	0.41%
Other	3350266	30.11%	2751391	32.46%	2889350	24.59%	2826275	29.89%	3840039	34.91%	4531407	36.58%

EV: Extracellular vesicle; hNSPC: human neural stem/progenitor cell; ID: inhibitor of DNA binding; WT: wild-type.

**Additional Table 2:** Differentially expressed miRNAs in EVs derived from ID3^-/-^ hNSPCs and WT hNSPCs

sRNA	KO_readcount	WT_readcount	log2FoldChange	pval	padj
hsa-let-7b-5p	10650.81043	5234.746283	1.024924432	0.003786	0.032984
hsa-let-7c-3p	88.84743927	20.51436228	2.112293433	0.006298	0.049897
hsa-miR-10b-3p	72.3197607	236.8867461	-1.707982301	0.002114	0.020814
hsa-miR-1180-3p	557.845497	258.4492451	1.110319034	0.000993	0.011835
hsa-miR-122-5p	34595.89996	3553.425449	3.283423152	4.64E-07	1.75E-05
hsa-miR-122b-3p	34595.89996	3553.425449	3.283423152	4.64E-07	1.75E-05
hsa-miR-1273h-3p	21.81108931	0.937204612	4.425897139	0.005512	0.045398
hsa-miR-128-3p	13784.58748	5522.079508	1.319930053	1.12E-05	0.000282
hsa-miR-130b-5p	1034.48812	357.6655242	1.533308331	0.003462	0.030747
hsa-miR-132-3p	45.56423613	538.6446412	-3.551467098	2.56E-16	1.16E-13
hsa-miR-132-5p	140.8459909	604.5950901	-2.095963654	1.71E-08	1.11E-06
hsa-miR-139-5p	65.39155241	203.5098244	-1.6299437	0.002717	0.026185
hsa-miR-140-3p	52.84369072	172.0130023	-1.710404027	0.003346	0.030315
hsa-miR-146a-5p	31.03821707	230.7948154	-2.90561694	2.58E-05	0.000557
hsa-miR-146b-5p	5596.03329	20210.17109	-1.852744645	1.89E-11	2.86E-09
hsa-miR-148a-3p	108187.2502	258305.082	-1.255540104	0.000182	0.002754
hsa-miR-148b-3p	961.6967599	2849.032775	-1.568072785	4.90E-05	0.000965
hsa-miR-152-3p	314.1663958	680.4135637	-1.116769024	0.00128	0.01326
hsa-miR-15b-5p	1.403388623	35.77483358	-4.529951579	0.00047	0.006256
hsa-miR-192-5p	529.0697901	187.8349202	1.496611611	0.001251	0.01326
hsa-miR-199a-3p	1935.865894	13905.15114	-2.844949761	6.33E-13	1.43E-10
hsa-miR-199a-5p	925.2426924	4978.130088	-2.428172171	4.42E-11	4.01E-09
hsa-miR-199b-5p	656.3591604	2920.125905	-2.154762707	3.53E-11	4.00E-09
hsa-miR-20b-5p	45.22285394	5.057016441	3.126352716	0.004965	0.042434
hsa-miR-214-3p	93.85718103	374.871192	-2.007629493	0.000147	0.002568
hsa-miR-214-5p	189.2260661	675.4287298	-1.83770521	8.35E-07	2.36E-05
hsa-miR-218-5p	1572.153745	5701.226986	-1.859227661	6.18E-07	2.13E-05
hsa-miR-219a-2-3p	155.8799415	35.72583035	2.132039685	0.000167	0.002707
hsa-miR-219b-5p	155.8799415	35.72583035	2.132039685	0.000167	0.002707
hsa-miR-26b-5p	468.8329335	1542.55117	-1.71909121	0.001288	0.01326
hsa-miR-301a-5p	0.897135317	25.92699061	-4.874623531	0.003242	0.029969
hsa-miR-30a-5p	1522.934247	2688.952194	-0.820418321	0.006162	0.049848
hsa-miR-3120-3p	189.2260661	678.6192136	-1.84448482	7.21E-07	2.18E-05
hsa-miR-3184-3p	3943.51853	1143.587936	1.786485668	0.000646	0.008133
hsa-miR-328-3p	745.4121055	204.1773202	1.868145887	0.000178	0.002754
hsa-miR-346	92.63600167	9.632807118	3.245930501	0.001537	0.015468
hsa-miR-363-3p	1302.052198	518.4252117	1.327835253	2.09E-05	0.000474
hsa-miR-423-5p	3943.51853	1143.587936	1.786485668	0.000646	0.008133
hsa-miR-424-3p	521.4924193	237.8029266	1.131991119	0.001256	0.01326
hsa-miR-4485-3p	128.4111763	24.29775537	2.408055355	3.42E-05	0.000703
hsa-miR-451a	429.5127868	40.22836733	3.42535315	9.49E-07	2.53E-05
hsa-miR-452-5p	10.60949557	68.79343838	-2.666643499	0.001244	0.01326
hsa-miR-483-3p	279.7334779	615.316924	-1.137029654	0.005075	0.042572
hsa-miR-486-3p	243.3532876	50.41304018	2.27832833	0.000318	0.004507
hsa-miR-486-5p	284.7602301	53.5848685	2.417729493	6.68E-05	0.00126
hsa-miR-500a-3p	21.28758831	180.1487006	-3.05302788	3.53E-07	1.60E-05
hsa-miR-500a-5p	8.536505271	76.07832096	-3.101457905	0.006389	0.049897
hsa-miR-504-5p	63.43794459	12.05443995	2.405653359	0.001147	0.01326
hsa-miR-532-5p	1349.613285	4233.406507	-1.649332422	2.70E-08	1.53E-06
hsa-miR-574-3p	63.58115531	443.3511685	-2.798360297	5.17E-09	3.90E-07
hsa-miR-671-3p	118.5142826	24.66456133	2.278269433	0.000127	0.002301
hsa-miR-675-3p	2.112544289	35.80899303	-4.069365691	0.000945	0.011568
hsa-miR-9-5p	53930.41177	22420.012	1.266283922	1.30E-05	0.000309
hsa-miR-92b-3p	3045.797342	578.2492886	2.397974121	6.58E-07	2.13E-05
hsa-miR-92b-5p	289.0966083	38.9954119	2.898823145	0.000203	0.002965
hsa-miR-95-3p	380.600055	81.92889349	2.212910935	1.73E-07	8.69E-06
hsa-miR-98-5p	2742.879567	7838.325669	-1.514894317	0.00043	0.005897
hsa-miR-99a-5p	224498.6406	125040.732	0.844308761	0.002838	0.026786

EV: Extracellular vesicle; hNSPC: human neural stem/progenitor cell; ID: inhibitor of DNA binding; WT: wild-type.

**Figure 6:**
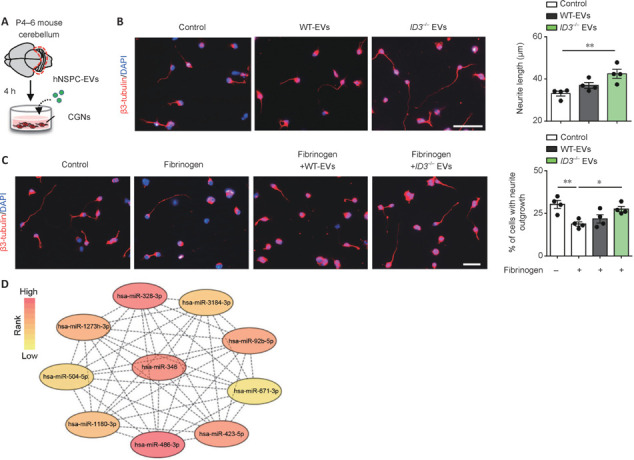
*ID3*-depleted hiPSC-derived NSPCs secrete beneficial EVs that support neuroregeneration. (A) Schematic of the hNSPC-EV treatment of mouse cerebellar granule neurons (CGNs) isolated on postnatal days 4 to 6. (B) Immunolabeling of β3-tubulin (red) in mouse CGNs left untreated or treated with EVs derived from WT or *ID3*^–/–^ hNSPCs. Quantification of the β3-tubulin^+^ neurite length (right) in untreated CGNs (control) or 24 hours after treatment with WT or *ID3*^–/–^ hNSPCs secreted EVs (*n* = 4 mice, a minimum of 350 neurons were analyzed per condition). (C) Immunolabeling of β3-tubulin (red) in mouse CGNs left untreated (control), treated with fibrinogen, or treated with fibrinogen in combination with EVs derived from WT or *ID3*^–/–^ hNSPCs. Quantification of the percentage of mouse CGNs bearing neurites (left) in untreated CGNs or 24 hours after fibrinogen treatment with EVs secreted from WT or *ID3*^–/–^ hNSPCs (*n* = 4 mice, a minimum of 200 neurons were analyzed per condition). Scale bars: 50 µm (B, C). Data are expressed as the mean ± SEM. One-way analysis of variance with Bonferroni’s multiple comparisons were performed, *P < 0.05, **P < 0.01. (D) Network analysis by cytoHubba, a Cytoscape plugin that implements eleven node ranking methods to evaluate the importance of nodes in a biological network, identified the top 10 core miRNAs that are enriched in the EVs of *ID3*^–/–^ hNSPCs. The miRNAs ranking from high to low are noted from red to yellow, respectively. ID: Inhibitor of DNA binding; hiPSC: human induced pluripotent stem cell; hNSPC: human neural stem/progenitor cell; NSPC: neural stem/progenitor cell; WT: wild-type.

Finally, in a first attempt to assess the potential of *ID3*^–/–^ hNSPCs to promote neurorepair, we treated primary mouse cerebellar granule neurons (CGNs) with EVs derived from either control or *ID3*^–/–^ hNSPCs (**Figure 6A**). Postnatal CGNs were previously used to identify inhibitors of axonal regeneration (Wang et al., 2002) and, importantly, to identify fibrinogen as an inhibitor of neurite outgrowth (Schachtrup et al., 2007). Remarkably, CGNs treated with EVs from *ID3*^–/–^ hNSPCs showed a **~**28% increase in neurite length compared with untreated CGNs and a **~**15% increase compared with CGNs treated with EVs from WT hNSPCs (**Figure 6B** and **Additional Figure 6A**). Moreover, treating CGNs with EVs from *ID3*^–/–^ hNSPCs rescued the fibrinogen-mediated inhibition of neurite outgrowth, resulting in a **~**47% increase in the number of CGNs extending neurites compared with fibrinogen-treated CGN controls (**Figure 6C** and **Additional Figure 6B**). Finally, KEGG pathway enrichment analysis revealed that genes targeted by the up-regulated miRNAs in the EVs of ID3-depleted hNSPCs are specifically involved in ‘Axon guidance’, ‘MAPK signaling pathway’ and ‘Protein kinases’ (**Additional Figure 6C**). The network composed of up-regulated miRNA was calculated by the Cytohubba (Chin et al., 2014), and top 10 miRNAs (e.g., has-miR-486-3p, hsa-miR-328-3p, and has-miR-486-3p) delineate core miRNAs in EVs of *ID3*^–/–^ hNSPCs that potentially synergistically promote neurogenesis and neurorepair (**Figure 6D**). Overall, our results demonstrate that fibrinogen alters the composition of the lesion environment after SCI and regulates hNSPC differentiation into astrocytes, while *ID3*^–/–^ hNSPCs resist astrogenic stimuli and promote neurite outgrowth with a distinctive miRNA profile in their secreted EVs.

## Discussion

The identification of extracellular environmental factors regulating the differentiation and functionality of hNSPCs in injury environments remains a fundamental and unresolved question.

Our results suggest that upon SCI and vascular damage, the blood-derived coagulation factor fibrinogen dramatically changes the host spinal-cord microenvironment at a subacute phase after SCI, potentially hindering NSPC transplantation therapy (Tetzlaff et al., 2011; Ahuja et al., 2020). Fibrinogen drives the differentiation of transplantable hNSPCs into astrocytes via a BMP-signaling-dependent mechanism. Our results suggest the following working model in traumatic CNS injury: (i) SCI leads to massive deposition of blood-derived fibrinogen, which creates a hostile microenvironment. (ii) Fibrinogen promotes hNSPC differentiation into reactive astrocytes via activation of the BMPRI signaling pathway and induction of inhibitor of DNA binding (ID) 3 expression. (iii) *ID3*^–/–^ hNSPCs, generated by CRISPR/Cas9-mediated genome editing, are resistant to astrogenic stimuli. Instead, these *ID3*^–/–^ hNSPCs secrete EVs that encase a distinguishable miRNA profile, enhancing neurite outgrowth. Thus, blood-derived extracellular fibrinogen deposition may regulate the functionality of transplanted hNSPCs, and targeting ID3 in hNSPCs might be a therapeutic strategy to create lesion-environment-resistant hNSPCs, potentially with the capacity to promote CNS repair.

Transplantation of iPSC-derived NSPCs represents one potential therapeutic strategy for replacing lost neural tissue and improving outcomes after SCI (Bradbury and Burnside, 2019; Katoh et al., 2019; Zheng and Tuszynski, 2023). Despite considerable progress in the transplantation of NSPCs capable of generating neurons and glia, the extent to which the host lesion environment modulates NSPC identity is poorly understood. Our data show that blood-derived fibrinogen creates an astrogenic milieu for transplantable NSPCs after SCI. Recent studies identified that non-cell autonomous cues in the injured spinal cord bias differentiation of transplanted NSPCs toward astrocytes (Khazaei et al., 2020; O’Shea et al., 2022). We demonstrated that fibrinogen deposition forms a provisional extracellular matrix that triggers immune responses, induces the deposition of inhibitory CSPGs after SCI, and directly regulates the differentiation of hNSPC into astrocytes *in vitro*. Importantly, in this study, we identify fibrinogen as a blood-derived extrinsic cue driving the cell identity of transplantable hNSPCs.

SCI-related environmental factors such as immune cell activation, extracellular CSPG deposition, and a lack of trophic support are known to trigger aberrant differentiation and impaired function in transplanted NSPCs (Bradbury and Burnside, 2019; Katoh et al., 2019; Zheng and Tuszynski, 2023). Mitigating the hostile microenvironment by either delivering neurotrophic factors or digesting chondroitinase ABC of CSPGs controls the identity of transplanted NSPCs and promotes neurorepair (Karimi-Abdolrezaee et al., 2010; Blesch et al., 2012; Suzuki et al., 2017). Transplanted NSPCs constitutively secrete neurotrophic factors to promote host axonal growth after SCI (Lu et al., 2003), and, furthermore, engineered NSPCs that express neurotrophins demonstrate the feasibility of a simultaneous gene and cell replacement approach (Park et al., 2006). Here, we showed that CRISPR/Cas9-modified hNSPCs were resistant to astrogenic stimuli and had a beneficial EV profile that promoted neurite outgrowth. Thus, engineering hNSPCs to achieve a desired NSPC identity to promote neurorepair might be a strategy to overcome limited regeneration after SCI. 

NSPCs promote regeneration predominantly through the release of bioactive molecules, such as growth factors, cytokines, and EVs (Dutta et al., 2021; Khalatbary, 2021; Yuan et al., 2021; Bonetto and Grilli, 2023; Zhang et al., 2023). In particular, NSPC-derived EVs have multiple therapeutic effects—such as neuroprotection, inhibition of apoptosis, and promotion of axonal regeneration—in various CNS diseases and injuries, including SCI (Dutta et al., 2021; Khalatbary, 2021; Zhang et al., 2023). Compared with EVs from, for example, cancer cells or mesenchymal stem cells, the content and functionality of hNSPC-derived EVs for neurorepair is largely unexplored. Promisingly, our data revealed that *ID3*^–/–^ hNSPC-derived EVs have an exclusive miRNA profile (e.g., let-7b, miR-9, miR-17-92, and miR-132). Notably, some of these miRNAs regulate NSPC differentiation and promote neurite outgrowth (Zhao et al., 2009; Volvert et al., 2012; Chen et al., 2018; Favaloro et al., 2022). While miR-92b-3p enhances neurite growth and facilitates functional recovery via the PTEN/AKT pathway in SCI (Chen et al., 2019), extracellular let-7b inhibits inflammation after SCI, in addition to promoting axonal growth (Liu et al., 2024). Our study demonstrated that administering EVs derived from *ID3*^–/–^ hNSPCs reversed the fibrinogen-induced inhibition of neurite outgrowth, indicating the potential role of these EVs in promoting neuroregeneration. Modification of the BMPRI–ID3 signaling pathway, therefore, endows transplantable hNSPCs with promising features for next-generation cell-free therapies via their differential expression of extracellular miRNAs that regulate neural differentiation, astrocyte activation, and potential immune responses.

NSPC grafts into CNS lesions form new neurons but are also biased towards astroglia-like differentiation and contribute to naturally occurring wound-repair processes (Khazaei et al., 2020; O’Shea et al., 2022). Here, we described how the blood-derived molecule fibrinogen functions as a key extracellular cue, instructing transplantable hNSPC differentiation toward astrocytes via the BMPRI–ID3 axis. EVs derived from NSPCs demonstrate low immunogenicity and can be developed efficiently. Id proteins are central to responses to environmental changes and increase in CNS injury and disease; cellular responses in adult NSPCs implicate Id proteins as prime candidates for manipulating stem cell behavior (Bohrer et al., 2015; Pous et al., 2020; Chu et al., 2021). As a first step, transplantation of *ID3*^–/–^ hNSPCs and their secreted EVs should be tested for long-term effects on functional behavior and repair after SCI in rodents, including tests for unwanted side effects, e.g. cell migration and teratogenicity (Hassan et al., 2024; Lin et al., 2025).

Since the fibrinogen-induced BMPRI–ID3 axis drives hNSPC identity (as demonstrated in our study), we suggest that manipulation of these fibrinogen-induced signaling pathways in human neural stem cells could tailor their fate and functions to promote CNS repair.

This study has some limitations. First, while we demonstrated that *ID3*^–/–^ hNSPCs exhibited resistance against astrogenic fibrinogen stimuli *in vitro*, future *in vivo* studies including transplanting these modified hNSPCs or their derived EVs into the injured spinal cord will provide more conclusive evidence for their reparative capacity. Second, our initial investigation into the neuroregenerative effects of *ID3*^–/–^ hNSPC-derived EVs was performed on CGNs and further studies should explore their efficacy across different neuronal populations.

In conclusion, deposition of blood-derived fibrinogen after SCI promotes the differentiation of transplantable hiPSC-derived NSPCs into reactive astrocytes via the BMPRI–ID3 axis. *ID3*^–/–^ hNSPC are resistant to astrogenic fibrinogen stimuli and secrete EVs with an altered miRNA profile that promotes neurite outgrowth. Collectively, these findings point to fibrinogen as a non-cell autonomous cue that directs transplantable NSPC identity and highlight the BMPRI–ID3 axis as a promising target for next-generation NSPC therapy after traumatic CNS injury.

## Additional files:

***Additional Figure 1:*** *Fibrinogen deposition induces scar formation and inflammation after SCI without affecting the lesion size or behavioral assessment.*

Additional Figure 1 Fibrinogen deposition induces scar formation and inflammation after SCI without affecting the lesion size or behavioral assessment.
(A) Immunolabeling of fibrinogen (red) in the spinal cord parenchyma 7 days after SCI. Quantification of fibrinogen immunoreactivity (IR) per lesion area (indicated by dashed lines) in fibrinogen-depleted mice compared with control animals (ancrod, n = 6 mice; NaCl, n = 5 mice) 7 days after SCI. (B) Immunolabeling of GFAP (green) and IBA-1 (magenta) in the spinal cord of fibrinogen-depleted mice (ancrod) and control animals (NaCl) 7 days after SCI. (C) Immunolabeling of NeuN (red) in spinal cord sections (injury epicentre, 1mm and 2 mm away from the epicentre) from fibrinogen-depleted (ancrod) and control (NaCl) mice 7 days after SCI. Dashed boxes indicate magnifications showing NeuN+ neurons in grey matter. Quantification of NeuN+ neurons in the spinal cord grey matter of fibrinogen-depleted mice compared with control animals (ancrod, n = 5 mice; NaCl, n = 5 mice) 7 days after SCI. (D) Representative luxol fast blue-stained spinal cord sections (injury epicentre, 1mm and 2 mm away from the epicentre) from fibrinogen-depleted (ancrod) and control (NaCl) mice 7 days after SCI (lesion area indicated by dashed lines). Quantification of the spared white matter area in the spinal cord of fibrinogen-depleted mice compared with control animals (ancrod, n = 6 mice; NaCl, n = 5 mice) 7 days post-SCI. (E) Assessment of locomotor recovery with the Basso Mouse Scale (BMS) 7 days after SCI in fibrinogen-depleted mice and control animals (ancrod, n = 6 mice; NaCl, n = 5 mice). Scale bars: 200 μm (A-C), 100 μm (C right), 400 μm (D). Data are the mean ± SEM. Unpaired Student’s *t*-tests (A-E) were performed. ***P < 0.001. DAPI: 4’,6-Diamidino-2-phenylindole; GFAP: glial fibrillary acidic protein; IBA-1: ionized calcium-binding adapter molecule 1; ns: not significant; SCI, spinal cord injury.

***Additional Figure 2:*** *Differentiation of caudalized hiPSC-derived NSPCs into astrocytes.*

Additional Figure 2 Differentiation of caudalized hiPSC-derived NSPCs into astrocytes.
(A) Representative images of different stages of astrocyte differentiation from hNSPCs. hiPSCs initially formed three-dimensional aggregates, the so-called embryoid bodies, in suspension culture. The embryoid bodies were differentiated to early neuroepithelial cells in the absence of exogenous growth factors for 10 days, followed by patterning with morphogens between days 10 and 21. To generate astrocytes, hNSPCs were differentiated for 5 days with ciliary neurotrophic factor. Astrocytes expressed typical markers, such as S100β (green) and GFAP (red). (B) Immunolabeling of spinal cord marker HOXB4 (red) and rostral brain marker OTX2 (green) in retinoid acid (RA)-treated or -untreated hNSPCs. Similar data were obtained for multiple batches (n = 3). (C) Relative expression of *ID3* mRNA in hiPSCs and in hNSPCs at different stages, as determined by quantitative PCR (n = 3). Scale bars: 200 μm (A, B). Data are the mean ± SEM. A one-way analysis of variance with Bonferroni’s multiple comparisons was performed. **P < 0.01. DAPI: 4’,6-Diamidino-2-phenylindole; hiPSC: human induced pluripotent stem cell; hNSPC: human neural stem/progenitor cell; ID: inhibitor of DNA binding; NSPC: neural stem/progenitor cell.

***Additional Figure 3:*** *Depleting ID3 in hiPSC-derived NSPCs via CRISPR/Cas9 ribonucleoprotein.*

Additional Figure 3 Depleting *ID3* in hiPSC-derived NSPCs via CRISPR/Cas9 ribonucleoprotein.
(A) T7 endonuclease I (T7E1) assay for analyzing the knockout efficiency of different gRNAs targeting the *ID3* locus. CRISPR/Cas9-edited hiPSCs were amplified by PCR and the resulting PCR amplicon digested by T7E1. The fraction of cleaved product is indicated. (B) Indel distribution was determined in CRISPR/Cas9-edited hiPSC polyclones that were edited with Cas9/gRNA2. The chart indicates the size and the percentages of insertions and deletions at the *ID3* target site. (C) Representative images of hiPSCs after limiting dilution for monoclonal selection and their recovery to a healthy clonal phenotype. (D) Indel distribution was determined in an *ID3*-edited hiPSC clone (clone#25). The chart indicates the size and the percentages of insertions and deletions at the target site. R^2^ reflects how well the indel distribution proposed by ICE fits the Sanger sequence data of the edited sample. (E) Genome integrity of the *ID3*-depleted hiPSC clone (clone#25) was evaluated and compared to the original hiPSC clone (WT) via digital PCR technology. (F) Representative PCR gel electrophoresis results showing the absence of a variety of Mycoplasma/Mollicutes strains in the cell culture of WT and *ID3*^-/-^ hiPSCs and caudalized hNSPCs. Ctrl: Control reaction without template. Scale bar: 100 μm (C). hiPSC: human induced pluripotent stem cell; hNSPC: human neural stem/progenitor cell; ICE: inference of CRISPR edits; ID: inhibitor of DNA binding; NSPC: neural stem/progenitor cell.

***Additional Figure 4:*** *ID3-depleted hiPSC-derived NSPCs are resistant to fibrinogen-induced astrogenesis.*

Additional Figure 4 *ID3*-depleted hiPSC-derived NSPCs are resistant to fibrinogen-induced astrogenesis.
(A) Representative images of Nestin+ (red, top) and CD44+ (red, bottom) glial progenitors in *ID3*^-/-^ hNSPCs 5 days after fibrinogen treatment (n = 2). (B) Immunolabeling of TUJ-1^+^ neurons (red) and GFAP^+^ astrocytes (green) in WT and *ID3*^-/-^ hNSPCs 5 days after fibrinogen treatment. Quantification of TUJ-1^+^ neurons in WT and *ID3*^-/-^ hNSPCs 5 days after fibrinogen treatment (n = 7). Scale bars: 200 μm (A-B). Data are the mean ± SEM. An unpaired Student’s *t*-test (B) was performed. **P < 0.01. DAPI: 4’,6-diamidino-2-phenylindole; GFAP: glial fibrillary acidic protein; hiPSC: human induced pluripotent stem cell; hNSPC: human neural stem/progenitor cell; ID: inhibitor of DNA binding; NSPC: neural stem/progenitor cell; TUJ-1: β-tubulin; WT: wild-type.

***Additional Figure 5:*** *Isolation and characterization of EVs released by WT and ID3-depleted hiPSC-derived NSPCs.*

Additional Figure 5 Isolation and characterization of EVs released by WT and *ID3*-depleted hiPSC-derived NSPCs.
(A) Schematic showing the isolation of EVs from hNSPC-conditioned medium using differential centrifugation and ultracentrifugation (100K) techniques. All EVs were obtained from medium conditioned for 24 h. (B) Immunoblot for EV markers (CD63 and ALIX), luminal protein (β-actin), and apolipoprotein B (APOB) for EVs secreted by hNSPCs (n = 4). (C) Particle size distributions of EVs secreted by WT (black) and *ID3*^-/-^ (green) hNSPCs (n = 3). (D) Median particle size (left) and particle concentration (right) of EVs secreted by WT and *ID3*^-/-^ hNSPCs (n = 3). Data are the mean ± SEM. Unpaired Student’s *t*-tests were performed. EV: Extracellular vesicle; hiPSC: human induced pluripotent stem cell; hNSPC: human neural stem/progenitor cell; ID: inhibitor of DNA binding; ns: not significant; NSPC: neural stem/progenitor cell; WT: wild-type.

***Additional Figure 6:*** *ID3-depleted hiPSC-derived NSPCs secrete EVs that support neurite outgrowth.*

Additional Figure 6 *ID3*-depleted hiPSC-derived NSPCs secrete EVs that support neurite outgrowth.
(A) Percentage of mouse cerebellar granule neurons (CGNs) bearing neurites in untreated CGNs (control) or after treatment with EVs secreted by WT or *ID3*^-/-^ hNSPCs (n = 4 mice, a minimum of 350 neurons were analyzed per condition). (B) Quantification of the β3-tubulin+ neurite length in untreated CGNs or after fibrinogen treatment with EVs secreted from WT or *ID3*^-/-^ hNSPCs (n = 4 mice, a minimum of 200 neurons were analyzed per condition). (C) Enriched Kyoto Encyclopedia of Genes and Genomes (KEGG) pathways targeted by miRNAs derived from EVs secreted by *ID3*^-/-^ hNSPCs compared to WT hNSPCs (n = 3). Data are the mean ± SEM. One-way ANOVAs with Bonferroni’s multiple comparisons were performed. EV: Extracellular vesicle; hiPSC: human induced pluripotent stem cell; hNSPC: human neural stem/progenitor cell; ID: inhibitor of DNA binding; ns: not significant; NSPC: neural stem/progenitor cell; WT: wild-type.

***Additional Table 1:*** *Annotation of extracellular small RNAs in EVs derived from ID3*^*–/–*^ *hNSPCs and WT hNSPCs.*

***Additional Table 2:*** *Differentially expressed miRNAs in EVs derived from ID3*^*–/–*^ *hNSPCs and WT hNSPCs.*

## Data Availability

*The data that support the findings of this study are available from the corresponding author CS (christian.schachtrup@anat.uni-freiburg.de) upon reasonable request.*

## References

[R1] Ahuja CS, Mothe A, Khazaei M, Badhiwala JH, Gilbert EA, van der Kooy D, Morshead CM, Tator C, Fehlings MG (2020). The leading edge: Emerging neuroprotective and neuroregenerative cell-based therapies for spinal cord injury. Stem Cells Transl Med.

[R2] Akassoglou K, Yu WM, Akpinar P, Strickland S (2002). Fibrin inhibits peripheral nerve remyelination by regulating Schwann cell differentiation. Neuron.

[R3] Assou S, Girault N, Plinet M, Bouckenheimer J, Sansac C, Combe M, Mianne J, Bourguignon C, Fieldes M, Ahmed E, Commes T, Boureux A, Lemaitre JM, De Vos J (2020). Recurrent genetic abnormalities in human pluripotent stem cells: definition and routine detection in culture supernatant by targeted droplet digital PCR. Stem Cell Reports.

[R4] Basso DM, Fisher LC, Anderson AJ, Jakeman LB, McTigue DM, Popovich PG (2006). Basso Mouse Scale for locomotion detects differences in recovery after spinal cord injury in five common mouse strains. J Neurotrauma.

[R5] Bian B, Zhao C, He X, Gong Y, Ren C, Ge L, Zeng Y, Li Q, Chen M, Weng C, He J, Fang Y, Xu H, Yin ZQ (2020). Exosomes derived from neural progenitor cells preserve photoreceptors during retinal degeneration by inactivating microglia. J Extracell Vesicles.

[R6] Blesch A, Fischer I, Tuszynski MH (2012). Gene therapy, neurotrophic factors and spinal cord regeneration. Handb Clin Neurol.

[R7] Bohrer C, Pfurr S, Mammadzada K, Schildge S, Plappert L, Hils M, Pous L, Rauch KS, Dumit VI, Pfeifer D, Dengjel J, Kirsch M, Schachtrup K, Schachtrup C (2015). The balance of Id3 and E47 determines neural stem/precursor cell differentiation into astrocytes. EMBO J.

[R8] Bonetto V, Grilli M (2023). Neural stem cell-derived extracellular vesicles: mini players with key roles in neurogenesis, immunomodulation, neuroprotection and aging. Front Mol Biosci.

[R9] Bradbury EJ, Burnside ER (2019). Moving beyond the glial scar for spinal cord repair. Nat Commun.

[R10] Budnik V, Ruiz-Canada C, Wendler F (2016). Extracellular vesicles round off communication in the nervous system. Nat Rev Neurosci.

[R11] Chen D, Hu S, Wu Z, Liu J, Li S (2018). The role of MiR-132 in regulating neural stem cell proliferation, differentiation and neuronal maturation. Cell Physiol Biochem.

[R12] Chen Z, Li Z, Jiang C, Jiang X, Zhang J (2019). MiR-92b-3p promotes neurite growth and functional recovery via the PTEN/AKT pathway in acute spinal cord injury. J Cell Physiol.

[R13] Chin CH, Chen SH, Wu HH, Ho CW, Ko MT, Lin CY (2014). cytoHubba: identifying hub objects and sub-networks from complex interactome. BMC Syst Biol 8 Suppl.

[R14] Chu YH, Lin JD, Nath S, Schachtrup C (2021). Id proteins: emerging roles in CNS disease and targets for modifying neural stemcell behavior. Cell Tissue Res.

[R15] Conforti P, Mezey S, Nath S, Chu YH, Malik SC, Martinez Santamaria JC, Deshpande SS, Pous L, Zieger B, Schachtrup C (2022). Fibrinogen regulates lesion border-forming reactive astrocyte properties after vascular damage. Glia.

[R16] Cuny GD, Yu PB, Laha JK, Xing X, Liu JF, Lai CS, Deng DY, Sachidanandan C, Bloch KD, Peterson RT (2008). Structure-activity relationship study of bone morphogenetic protein (BMP) signaling inhibitors. Bioorg Med Chem Lett.

[R17] Deinsberger J, Reisinger D, Weber B (2020). Global trends in clinical trials involving pluripotent stem cells: a systematic multi-database analysis. NPJ Regen Med.

[R18] Donnelly DJ, Popovich PG (2008). Inflammation and its role in neuroprotection, axonal regeneration and functional recovery after spinal cord injury. Exp Neurol.

[R19] Dutta D, Khan N, Wu J, Jay SM (2021). Extracellular vesicles as an emerging frontier in spinal cord injury pathobiology and therapy. Trends Neurosci.

[R20] Favaloro F, DeLeo AM, Delgado AC, Doetsch F (2022). miR-17 approximately 92 exerts stage-specific effects in adult V-SVZ neural stem cell lineages. Cell Rep.

[R21] Grade S, Thomas J, Zarb Y, Thorwirth M, Conzelmann KK, Hauck SM, Gotz M (2022). Brain injury environment critically influences the connectivity of transplanted neurons. Sci Adv.

[R22] Hammad R, Alzubi J, Rhiel M, Chmielewski KO, Mosti L, Rositzka J, Heugel M, Lawrenz J, Pennucci V, Glaser B, Fischer J, Schambach A, Moritz T, Lachmann N, Cornu TI, Mussolino C, Schafer R, Cathomen T (2024). CRISPR-Cas12a for highly efficient and marker-free targeted integration in human pluripotent stem cells. Int J Mol Sci.

[R23] Hassan OI, Takamiya S, Asgarihafshejani A, Fehlings MG (2024). Bridging the gap: a translational perspective in spinal cord injury. Exp Biol Med (Maywood).

[R24] Karimi-Abdolrezaee S, Eftekharpour E, Wang J, Schut D, Fehlings MG (2010). Synergistic effects of transplanted adult neural stem/progenitor cells, chondroitinase, and growth factors promote functional repair and plasticity of the chronically injured spinal cord. J Neurosci.

[R25] Katoh H, Yokota K, Fehlings MG (2019). Regeneration of spinal cord connectivity through stem cell transplantation and biomaterial scaffolds. Front Cell Neurosci.

[R26] Khalatbary AR (2021). Stem cell-derived exosomes as a cell free therapy against spinal cord injury. Tissue Cell.

[R27] Khazaei M, Ahuja CS, Nakashima H, Nagoshi N, Li L, Wang J, Chio J, Badner A, Seligman D, Ichise A, Shibata S, Fehlings MG (2020). GDNF rescues the fate of neural progenitor grafts by attenuating Notch signals in the injured spinal cord in rodents. Sci Transl Med.

[R28] Krencik R, Zhang SC (2011). Directed differentiation of functional astroglial subtypes from human pluripotent stem cells. Nat Protoc.

[R29] Lien BV, Tuszynski MH, Lu P (2019). Astrocytes migrate from human neural stem cell grafts and functionally integrate into the injured rat spinal cord. Exp Neurol.

[R30] Lin M, Alimerzaloo F, Wang X, Alhalabi O, Krieg SM, Skutella T, Younsi A (2025). Harnessing stem cell-derived exosomes: a promising cell-free approach for spinal cord injury. Stem Cell Res Ther.

[R31] Liu J, Gaj T, Yang Y, Wang N, Shui S, Kim S, Kanchiswamy CN, Kim JS, Barbas CF (2015). Efficient delivery of nuclease proteins for genome editing in human stem cells and primary cells. Nat Protoc.

[R32] Liu J, Kong G, Lu C, Wang J, Li W, Lv Z, Tong J, Liu Y, Xiong W, Li H, Fan J (2024). IPSC-NSCs-derived exosomal let-7b-5p improves motor function after spinal cord Injury by modulating microglial/macrophage pyroptosis. J Nanobiotechnology.

[R33] Liu Y, Han SS, Wu Y, Tuohy TM, Xue H, Cai J, Back SA, Sherman LS, Fischer I, Rao MS (2004). CD44 expression identifies astrocyte-restricted precursor cells. Dev Biol.

[R34] Losurdo M, Grilli M (2020). Extracellular vesicles, influential players of intercellular communication within adult neurogenic niches. Int J Mol Sci.

[R35] Love MI, Huber W, Anders S (2014). Moderated estimation of fold change and dispersion for RNA-seq data with DESeq2. Genome Biol.

[R36] Lu P, Jones LL, Snyder EY, Tuszynski MH (2003). Neural stem cells constitutively secrete neurotrophic factors and promote extensive host axonal growth after spinal cord injury. Exp Neurol.

[R37] Lu P, Woodruff G, Wang Y, Graham L, Hunt M, Wu D, Boehle E, Ahmad R, Poplawski G, Brock J, Goldstein LS, Tuszynski MH (2014). Long-distance axonal growth from human induced pluripotent stem cells after spinal cord injury. Neuron.

[R38] Malik SC, Chu YH, Schachtrup C (2023). Pointing fingers at blood contact: mechanisms of subventricular zone neural stem cell differentiation. Neural Regen Res.

[R39] Martins-Macedo J, Lepore AC, Domingues HS, Salgado AJ, Gomes ED, Pinto L (2021). Glial restricted precursor cells in central nervous system disorders: Current applications and future perspectives. Glia.

[R40] Mathieu M, Nevo N, Jouve M, Valenzuela JI, Maurin M, Verweij FJ, Palmulli R, Lankar D, Dingli F, Loew D, Rubinstein E, Boncompain G, Perez F, Thery C (2021). Specificities of exosome versus small ectosome secretion revealed by live intracellular tracking of CD63 and CD9. Nat Commun.

[R41] Mendiola AS (2023). Defining blood-induced microglia functions in neurodegeneration through multiomic profiling. Nat Immunol.

[R42] Morton MC, Neckles VN, Feliciano DM (2019). Isolation of extracellular vesicles from subventricular zone neural stem cells. Methods Mol Biol.

[R43] Morton MC, Neckles VN, Seluzicki CM, Holmberg JC, Feliciano DM (2018). Neonatal subventricular zone neural stem cells release extracellular vesicles that act as a microglial morphogen. Cell Rep.

[R44] O’Shea TM, Ao Y, Wang S, Wollenberg AL, Kim JH, Ramos Espinoza RA, Czechanski A, Reinholdt LG, Deming TJ, Sofroniew MV (2022). Lesion environments direct transplanted neural progenitors towards a wound repair astroglial phenotype in mice. Nat Commun.

[R45] Ogawa Y, Sawamoto K, Miyata T, Miyao S, Watanabe M, Nakamura M, Bregman BS, Koike M, Uchiyama Y, Toyama Y, Okano H (2002). Transplantation of in vitro-expanded fetal neural progenitor cells results in neurogenesis and functional recovery after spinal cord contusion injury in adult rats. J Neurosci Res.

[R46] Park KI, Himes BT, Stieg PE, Tessler A, Fischer I, Snyder EY (2006). Neural stem cells may be uniquely suited for combined gene therapy and cell replacement: Evidence from engraftment of Neurotrophin-3-expressing stem cells in hypoxic-ischemic brain injury. Exp Neurol.

[R47] Percie du Sert N (2020). The ARRIVE guidelines 2.0: Updated guidelines for reporting animal research. PLoS Biol.

[R48] Peruzzotti-Jametti L (2021). Neural stem cells traffic functional mitochondria via extracellular vesicles. PLoS Biol.

[R49] Petersen MA (2017). Fibrinogen activates BMP signaling in oligodendrocyte progenitor cells and inhibits remyelination after vascular damage. Neuron.

[R50] Petersen MA, Ryu JK, Akassoglou K (2018). Fibrinogen in neurological diseases: mechanisms, imaging and therapeutics. Nat Rev Neurosci.

[R51] Popovich PG, Horner PJ, Mullin BB, Stokes BT (1996). A quantitative spatial analysis of the blood-spinal cord barrier. I. Permeability changes after experimental spinal contusion injury. Exp Neurol.

[R52] Pous L, Deshpande SS, Nath S, Mezey S, Malik SC, Schildge S, Bohrer C, Topp K, Pfeifer D, Fernandez-Klett F, Doostkam S, Galanakis DK, Taylor V, Akassoglou K, Schachtrup C (2020). Fibrinogen induces neural stem cell differentiation into astrocytes in the subventricular zone via BMP signaling. Nat Commun.

[R53] Raponi E, Agenes F, Delphin C, Assard N, Baudier J, Legraverend C, Deloulme JC (2007). S100B expression defines a state in which GFAP-expressing cells lose their neural stem cell potential and acquire a more mature developmental stage. Glia.

[R54] Schachtrup C, Lu P, Jones LL, Lee JK, Lu J, Sachs BD, Zheng B, Akassoglou K (2007). Fibrinogen inhibits neurite outgrowth via beta 3 integrin-mediated phosphorylation of the EGF receptor. Proc Natl Acad Sci U S A.

[R55] Schachtrup C, Ryu JK, Helmrick MJ, Vagena E, Galanakis DK, Degen JL, Margolis RU, Akassoglou K (2010). Fibrinogen triggers astrocyte scar formation by promoting the availability of active TGF-beta after vascular damage. J Neurosci.

[R56] Schachtrup C, Ryu JK, Mammadzada K, Khan AS, Carlton PM, Perez A, Christian F, Le Moan N, Vagena E, Baeza-Raja B, Rafalski V, Chan JP, Nitschke R, Houslay MD, Ellisman MH, Wyss-Coray T, Palop JJ, Akassoglou K (2015). Nuclear pore complex remodeling by p75(NTR) cleavage controls TGF-β signaling and astrocyte functions. Nat Neurosci.

[R57] Schachtrup C (2022). Modulating scar formation for improving brain repair: from coagulation and inflammation to cell therapy. Cell Tissue Res.

[R58] Shannon P, Markiel A, Ozier O, Baliga NS, Wang JT, Ramage D, Amin N, Schwikowski B, Ideker T (2003). Cytoscape: a software environment for integrated models of biomolecular interaction networks. Genome Res.

[R59] Shi Y, Inoue H, Wu JC, Yamanaka S (2017). Induced pluripotent stem cell technology: a decade of progress. Nat Rev Drug Discov.

[R60] Sliwinski C, Nees TA, Puttagunta R, Weidner N, Blesch A (2018). Sensorimotor activity partially ameliorates pain and reduces nociceptive fiber density in the chronically injured spinal cord. J Neurotrauma.

[R61] Sridhar A, Ohlemacher SK, Langer KB, Meyer JS (2016). Robust differentiation of mRNA-reprogrammed human induced pluripotent stem cells toward a retinal lineage. Stem Cells Transl Med.

[R62] Sullivan S (2018). Quality control guidelines for clinical-grade human induced pluripotent stem cell lines. Regen Med.

[R63] Suzuki H, Ahuja CS, Salewski RP, Li L, Satkunendrarajah K, Nagoshi N, Shibata S, Fehlings MG (2017). Neural stem cell mediated recovery is enhanced by Chondroitinase ABC pretreatment in chronic cervical spinal cord injury. PLoS One.

[R64] Tetzlaff W, Okon EB, Karimi-Abdolrezaee S, Hill CE, Sparling JS, Plemel JR, Plunet WT, Tsai EC, Baptiste D, Smithson LJ, Kawaja MD, Fehlings MG, Kwon BK (2011). A systematic review of cellular transplantation therapies for spinal cord injury. J Neurotrauma.

[R65] Thery C (2018). Minimal information for studies of extracellular vesicles 2018 (MISEV2018): a position statement of the International Society for Extracellular Vesicles and update of the MISEV2014 guidelines. J Extracell Vesicles.

[R66] van Niel G, Carter DRF, Clayton A, Lambert DW, Raposo G, Vader P (2022). Challenges and directions in studying cell-cell communication by extracellular vesicles. Nat Rev Mol Cell Biol.

[R67] Volvert ML, Rogister F, Moonen G, Malgrange B, Nguyen L (2012). MicroRNAs tune cerebral cortical neurogenesis. Cell Death Differ.

[R68] Wang KC, Kim JA, Sivasankaran R, Segal R, He Z (2002). P75 interacts with the Nogo receptor as a co-receptor for Nogo, MAG and OMgp. Nature.

[R69] Willis CM, Nicaise AM, Hamel R, Pappa V, Peruzzotti-Jametti L, Pluchino S (2020). Harnessing the neural stem cell secretome for regenerative neuroimmunology. Front Cell Neurosci.

[R70] Yu G, Wang LG, Han Y, He QY (2012). clusterProfiler: an R package for comparing biological themes among gene clusters. OMICS.

[R71] Yuan P, Ding L, Chen H, Wang Y, Li C, Zhao S, Yang X, Ma Y, Zhu J, Qi X, Zhang Y, Xia X, Zheng JC (2021). Neural stem cell-derived exosomes regulate neural stem cell differentiation through miR-9-Hes1 axis. Front Cell Dev Biol.

[R72] Zhang K, Yang Y, Ge H, Wang J, Lei X, Chen X, Wan F, Feng H, Tan L (2022). Neurogenesis and proliferation of neural stem/progenitor cells conferred by artesunate via FOXO3a/p27Kip1 axis in mouse stroke model. Mol Neurobiol.

[R73] Zhang X, Jiang W, Lu Y, Mao T, Gu Y, Ju D, Dong C (2023). Exosomes combined with biomaterials in the treatment of spinal cord injury. Front Bioeng Biotechnol.

[R74] Zhang Y, Sloan SA, Clarke LE, Caneda C, Plaza CA, Blumenthal PD, Vogel H, Steinberg GK, Edwards MS, Li G, Duncan JA, Cheshier SH, Shuer LM, Chang EF, Grant GA, Gephart MG, Barres BA (2016). Purification and characterization of progenitor and mature human astrocytes reveals transcriptional and functional differences with mouse. Neuron.

[R75] Zhao C, Sun G, Li S, Shi Y (2009). A feedback regulatory loop involving microRNA-9 and nuclear receptor TLX in neural stem cell fate determination. Nat Struct Mol Biol.

[R76] Zheng B, Tuszynski MH (2023). Regulation of axonal regeneration after mammalian spinal cord injury. Nat Rev Mol Cell Biol.

